# Fine-tuning of the flowering time control in winter barley: the importance of *HvOS2* and *HvVRN2* in non-inductive conditions

**DOI:** 10.1186/s12870-019-1727-9

**Published:** 2019-03-25

**Authors:** Arantxa Monteagudo, Ernesto Igartua, Bruno Contreras-Moreira, M. Pilar Gracia, Javier Ramos, Ildikó Karsai, Ana M. Casas

**Affiliations:** 1 Aula Dei Experimental Station (EEAD-CSIC), Avda. Montañana 1005, E-50059 Zaragoza, Spain; 2 Fundación ARAID, Zaragoza, Spain; 3 Centre for Agricultural Research, Hungarian Academy of Sciences, Martonvásár, H-2462 Hungary

**Keywords:** Barley, Gene expression, *HvCO2*, *HvFT3*, *HvOS2*, *HvVRN1*, *HvVRN2*, Photoperiod, *PPD-H1*, Vernalization

## Abstract

**Background:**

In winter barley plants, vernalization and photoperiod cues have to be integrated to promote flowering. Plant development and expression of different flowering promoter (*HvVRN1*, *HvCO2*, *PPD-H1*, *HvFT1*, *HvFT3*) and repressor (*HvVRN2*, *HvCO9* and *HvOS2*) genes were evaluated in two winter barley varieties under: (1) natural increasing photoperiod, without vernalization, and (2) under short day conditions in three insufficient vernalization treatments. These challenging conditions were chosen to capture non-optimal and natural responses, representative of those experienced in the Mediterranean area.

**Results:**

In absence of vernalization and under increasing photoperiods, *HvVRN2* expression increased with day-length, mainly between 12 and 13 h photoperiods in our latitudes. The flowering promoter gene in short days, *HvFT3*, was only expressed after receiving induction of cold or plant age, which was associated with low transcript levels of *HvVRN2* and *HvOS2*. Under the sub-optimal conditions here described, great differences in development were found between the two winter barley varieties used in the study. Delayed development in ‘Barberousse’ was associated with increased expression levels of *HvOS2*. Novel variation for *HvCO9* and *HvOS2* is reported and might explain such differences.

**Conclusions:**

The balance between the expression of flowering promoters and repressor genes regulates the promotion towards flowering or the maintenance of the vegetative state. *HvOS2*, an ortholog of *FLC*, appears as a strong candidate to mediate in the vernalization response of barley. Natural variation found would help to exploit the plasticity in development to obtain better-adapted varieties for current and future climate conditions.

**Electronic supplementary material:**

The online version of this article (10.1186/s12870-019-1727-9) contains supplementary material, which is available to authorized users.

## Background

Tight coordination of flowering time to environmental conditions is crucial for crop reproductive success and has a major impact on yield [1, 2]. Barley (*Hordeum vulgare* L.) and wheat (*Triticum* spp.) are long-day (LD) plants, flowering earlier under increasing day-lengths. Depending on their growth habit, cereals are classified as winter or spring. Winter cereals need a period of exposure to low temperature (vernalization), which must be completed in a timely manner so the plant is prepared to take full advantage of the induction of flowering by long days [3]. This requirement could make winter cereals more susceptible to climate change, since the probability of accumulating enough cold hours will likely decrease in warming winters. Winter barley varieties are sown in autumn, benefiting from the warmth of the soils and the humidity from autumn rains, which are essential at the beginning of the cycle. In the Mediterranean region, they have to survive a range of mild to harsh winters, and then flower sufficiently early in the spring to avoid the heat and drought of late spring or early summer. Barley ideotypes for future climatic conditions in Europe must present combinations of vernalization requirement and photoperiod responses tuned to the needs of each specific region [4]. For this reason, plant breeding for upcoming conditions demands comprehensive studies on the effect of photoperiod on major flowering genes, and their interaction with the vernalization pathway. In this regard, special emphasis should be given to environmental conditions closer to natural ones, as it is not known “whether the current model of photoperiodic flowering regulation can recapitulate the seasonal flowering mechanisms in complicated natural LD environments” [5].

The accepted gene model for vernalization-responsive varieties establishes that, during winter, cold exposure upregulates the floral promoter *HvVRN1*, which is required to downregulate the flowering repressor *HvVRN2*, allowing expression of the flowering inducer *HvFT1* in leaves [6]. *HvVRN2,* a *ZCCT-H* gene, is member of the *CONSTANS*-like gene family, which delays flowering until plants have satisfied their cold needs [7]. Winter barleys have the dominant variant, whose expression is highly dependent on day-length, being induced in long days [8, 9]. *HvVRN1* encodes an *AP1*-like MADS-box transcription factor [10–12]. It presents several alleles as a result of deletions or insertions in the first intron, associated with different degrees of vernalization requirement [13]. In winter barley, *HvVRN1* is expressed after exposure to low-temperatures [14, 15], although it can be activated by other pathways such as the developmental pathway, with a marked delay compared with induction by vernalization [9]. Induction of *HvVRN1* is related to changes in the pattern of histone methylation, whose maintenance provides a memory of cold exposure in winter barley plants [16]. This general mechanism is well established; however, important questions remain open. For instance, what are the precise environmental cues that govern the dynamics of this process? A second open question is which additional genes may play important roles in the vernalization pathway. In this respect, Bouché et al. [17] remarked that much remains to be learned about this process, including identifying additional components, beyond the *VRN1*/*VRN2* system. Indeed, there are phenotypic differences in vernalization effect among winter cultivars sharing *HvVRN1*/*HvVRN2* haplotypes that are still unexplained [18]. For instance, it has been suggested that additional genes may be acting as regulators of *VRN2* when exposed to cold [19, 20]. Some genes are good candidates to play a role in the vernalization pathway, like *ODDSOC2* (in barley, *HvOS2*), the monocot ortholog of *Arabidopsis thaliana FLOWERING LOCUS C* (*FLC*). This gene is a flowering repressor also downregulated by vernalization in barley [21] and *Brachypodium distachyon* [22], probably caused by binding of VRN1 to its promoter region [23].

*HvFT3*, a FT-like member of the PEBP family, and candidate gene for *PPD-H2* [24, 25], it was described as a promoter of flowering under short days (SD) in winter cultivars [26, 27], particularly under Mediterranean conditions [28–30]. Its role has been recently clarified specifically controlling spikelet initiation, but not floral development [31].

The photoperiod response regulator gene *PPD-H1*, also known as *HvPRR37* [32] determines the sensitivity to LD [33], and accelerates flowering mediating the induction of *HvFT1,* in winter cultivars after vernalization is fulfilled. There is also evidence of the involvement of several members of the family of *CONSTANS*-like genes in the vernalization and photoperiod pathways. *CO1* and *CO2* are LD-flowering promoters modulated by circadian clock and day-length [34, 35]. In wheat, CO2 competes with VRN2 to bind the NF-Y proteins, in a mechanism to integrate environmental cues through regulation of *HvFT1* [36]. Another member of this family, *HvCO9* (or *HvCMF11* in Cockram et al. [37]) is a paralog of *HvVRN2* [38], and has been identified as a negative regulator of flowering, whose expression has been reported under non-inductive SD conditions [39].

This study focuses on the identification of factors (genes and environmental conditions) responsible for repression of flowering in winter barley. Previous studies have demonstrated that *HvVRN2* expression needs induction by long days [9], but the exact day-length that triggers this gene is unknown, as most studies have been performed in growth chambers, under fixed photoperiods. This question is relevant from the agronomic point of view. Song et al. [5] highlighted the importance of optimizing controlled conditions to reflect closely the natural environments. Thus, our approach was addressed trying to mimic the photoperiod conditions in natural Mediterranean environments. We hypothesize that there is a vernalization window for satisfying the cold requirement, in order to make the plant competent to flower at the right time and achieve a good yield. In the experiments presented here, the first objective was to determine the day-length threshold leading to induction of the repressor *HvVRN2*. A second objective was to characterize further the role of other possible inducers and repressors of flowering under incomplete or null vernalization. We investigate the effects of photoperiod on the transcript levels of selected genes in winter barley, by examining photoperiod responses in the medium-long term (21–90 days) in two winter cultivars, ‘Hispanic’ and ‘Barberousse’, that present different adaptation patterns [40, 41]. Our final aim was to provide new information on the complex mechanism of flowering in suboptimal conditions, to facilitate breeding for present and future climate conditions.

## Results

Both ‘Hispanic’ and ‘Barberousse’ responded to vernalization, with a marked acceleration of development as the cold period applied increased from 0 to 45 days with day-length of 16 h (Additional file 1: Figure S1). There were also overall differences in earliness.

### Gene expression under increasing natural photoperiod

**Fig. 1:**
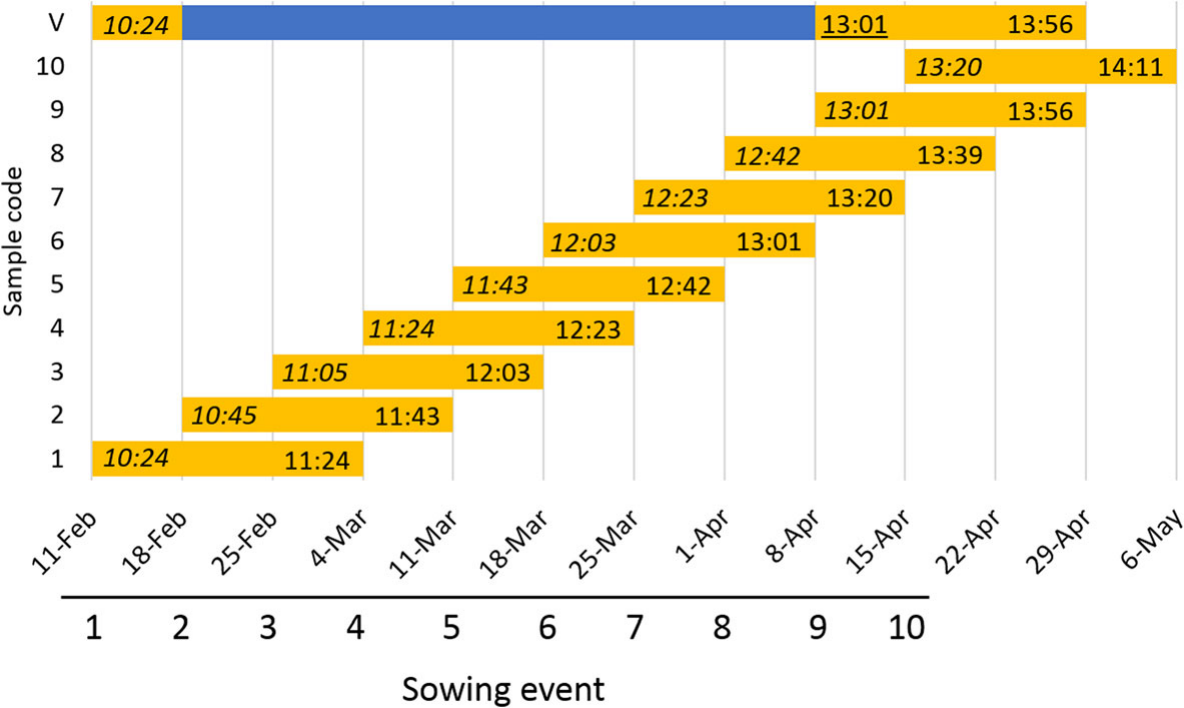
Experiment planning. Each sowing and its sampling are represented. Yellow bars show the time that plants were kept under non-vernalized conditions. Blue bar shows the time spent in the vernalization chamber. X-axis represent dates of start of experiment and sampling date (three weeks after sowing). The second numbers inside the yellow bars are the day-length at sampling date (HH:MM). The first numbers in italics represent day-length at sowing day, and underlined numbers are day-length in the shift day (vernalized plants were transferred to glasshouse). Sunrise and sunset are the times when the upper edge of the Sun’s disc touches the horizon

In order to determine the day-length threshold that induces the expression of *HvVRN2*, experiment 1 involved sequential sowings in a greenhouse, one week apart. Natural day-length at sampling (for 21-day-old plants) increased from ~ 11 h 30 min at the first sowing to ~ 14 h at the 9th sowing event, and also for the VER control (Fig. 1).

**Fig. 2:**
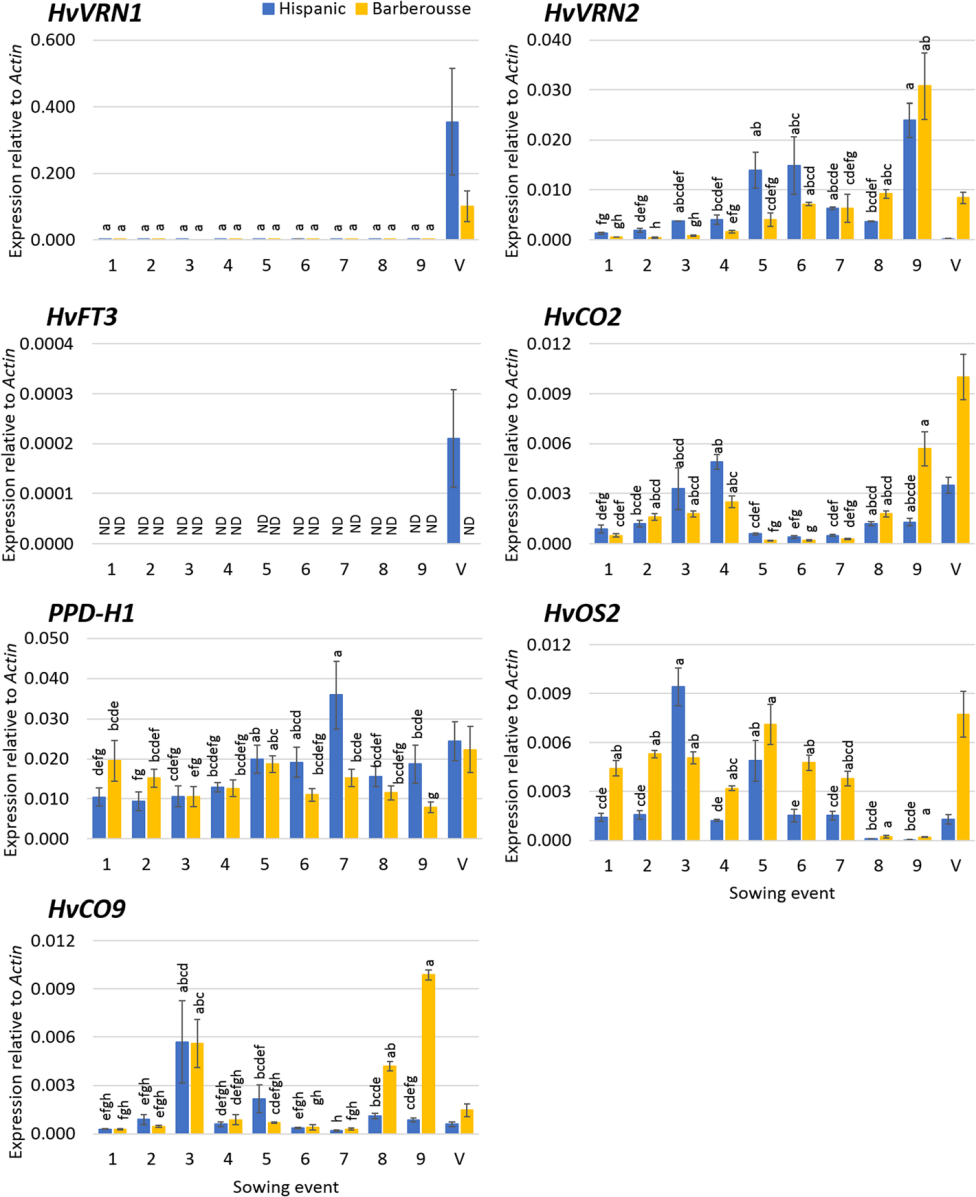
Gene expression three weeks after sowing. X-axis represent the successive sowings, from 11th February until 8th April. Unvernalized plants (sowings 1 to 9) and vernalized control (V) of ‘Hispanic’ (blue) and ‘Barberousse’ (yellow) are plotted. Mean of 3 biological replicates. Error bars are SEM. ND, Not detected. *HvFT1* expression is not reported as it was null for all non-vernalized samples. For each NV time-point, bars with the same letter are not significantly different at *P* < 0.05 according to ANOVA that included genotypes and all sampling times

Surprisingly, expression of *HvVRN2* was detected at all time points, in plants both 14 and 21 days old. It was low in the first sowings, which were grown under shorter photoperiods (Fig. 2). Between 12 and 13 h photoperiods, corresponding to the end of March in our latitude, the levels of *HvVRN2* increased in both genotypes. At 21 days, there were significant differences in *HvVRN2* expression between genotypes and sowings, without interaction between them (Additional file 1: Table S1), indicating similar pattern of responses across genotypes. A contrast between the four earliest sowings (1–4) vs the five latest (5–9) explained as much as 78% of the variation between sowings, rendering the remaining variance (genotypes by sampling point, within day-lengths groups), non-significant (Additional file 1: Table S2A). Therefore, the surge in expression between sowings 4 and 5 is the main factor affecting *HvVRN2* expression for both genotypes. This same trend was also detected in 14-day-old plants, with slightly lower expression values overall (Additional file 1: Figure S2).

Expression of *HvCO2* increased in both genotypes up to sowing 4. Then, it decreased to very low levels, not rising again until sowing 8 and 9. The main change in expression patterns occurred again between sowings 4 and 5, for both genotypes (Additional file 1: Table S2B).

*HvVRN1* expression was detected only in VER plants (Fig. 2), and *HvFT1* was not detected in any sample at this stage (data not shown). Without vernalization, neither genotype showed expression of *HvFT3* (Fig. 2). This was expected for ‘Barberousse’, as it has the null allele, but we did not anticipate this result for ‘Hispanic’. In this genotype, the expression levels were below the detection limit, except for VER plants.

In general, ‘Barberousse’ presented higher *HvOS2* expression levels than ‘Hispanic’ (Additional file 1: Table S1), except for the last samplings, when *HvOS2* expression was barely detectable in both genotypes (Fig. 2). Expression of *HvCO9* was low and variable, with no observable trends for any genotype. *PPD-H1* expression showed fluctuations in expression apparently independent from genotypes.

At the end of the experiment (May 19th, with 15 h of light), the number of apices at reproductive stage per plant was recorded (Additional file 1: Figure S3). ‘Hispanic’ plants were more developed than ‘Barberousse’s. Among NV plants, only the second sowing event of ‘Hispanic’ reached the stage Z49 (first awns visible) at the end of the experiment (83 days after sowing). No data were available for the first sowing at that moment, as plants were dissected earlier, again with only ‘Hispanic’ showing reproductive apices after 72 days. At termination, VER ‘Hispanic’ and ‘Barberousse’ plants also showed apices at reproductive stage, ‘Barberousse’ more delayed than ‘Hispanic’.

**Fig. 3:**
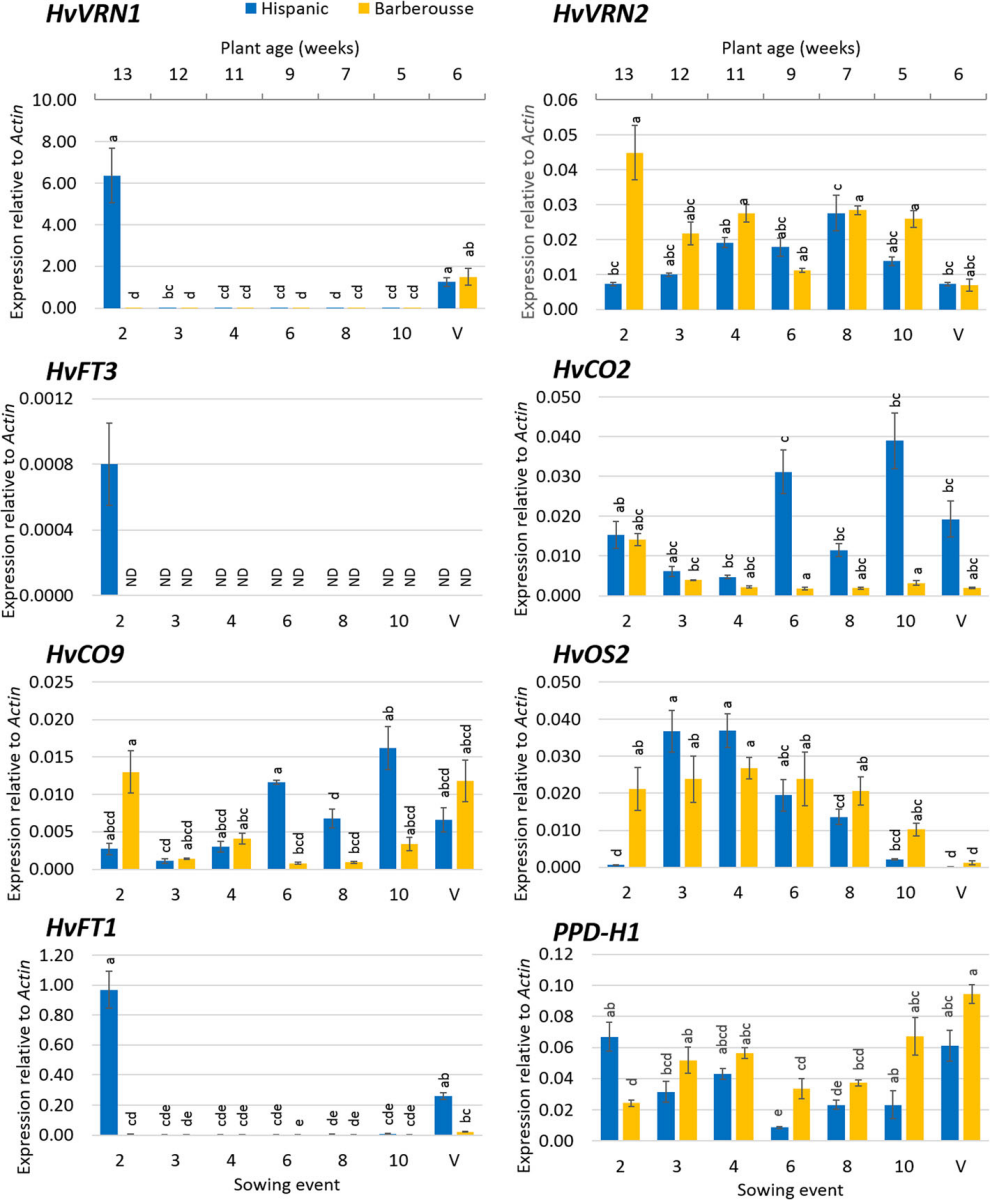
Cross-sectional gene expression under 15 h of natural daylight of the sequential sowings under natural photoperiod experiment. X- upper axis represent the weeks after sowing of unvernalized plants. Control plants (V) were maintained under natural photoperiod for 6 weeks after 49 days of vernalization. Mean of 3 biological replicates. Error bars are SEM. For each sampling time-point and genotype, bars with the same letter are not significantly different at *P* < 0.05 according to ANOVA that included all factors

Expression levels on this same date were analysed (Fig. 3, Additional file 1: Table S3), across all sowings. For all NV plants, flowering promoters (*HvVRN1*, *HvFT1* and *HvFT3*) were induced only in ‘Hispanic’ oldest plants, at the first point available (sowing event 2), and were not expressed in ‘Barberousse’, in full accordance with apex development. Concurrently, in plants from sowing 2, repressors *HvVRN2* and *HvOS2* were down-regulated in ‘Hispanic’, and induced in ‘Barberousse’.

### Gene expression affected by plant age and length of vernalization treatment

Experiment 1 made evident that gene expression was dependent on the plant’s developmental stage (Fig. 3). Therefore, for some genes, induction was dependent on plant age. A second experiment was conceived, to assess the relevance of other factors on gene expression, namely day-length, plant age and degree of vernalization. Thus, we set the day-length at 12 h, representative of day-length around the start of stem elongation in natural conditions in our region, and short enough not to elicit LD responses. This was combined with increasing yet insufficient vernalization.

**Fig. 4:**
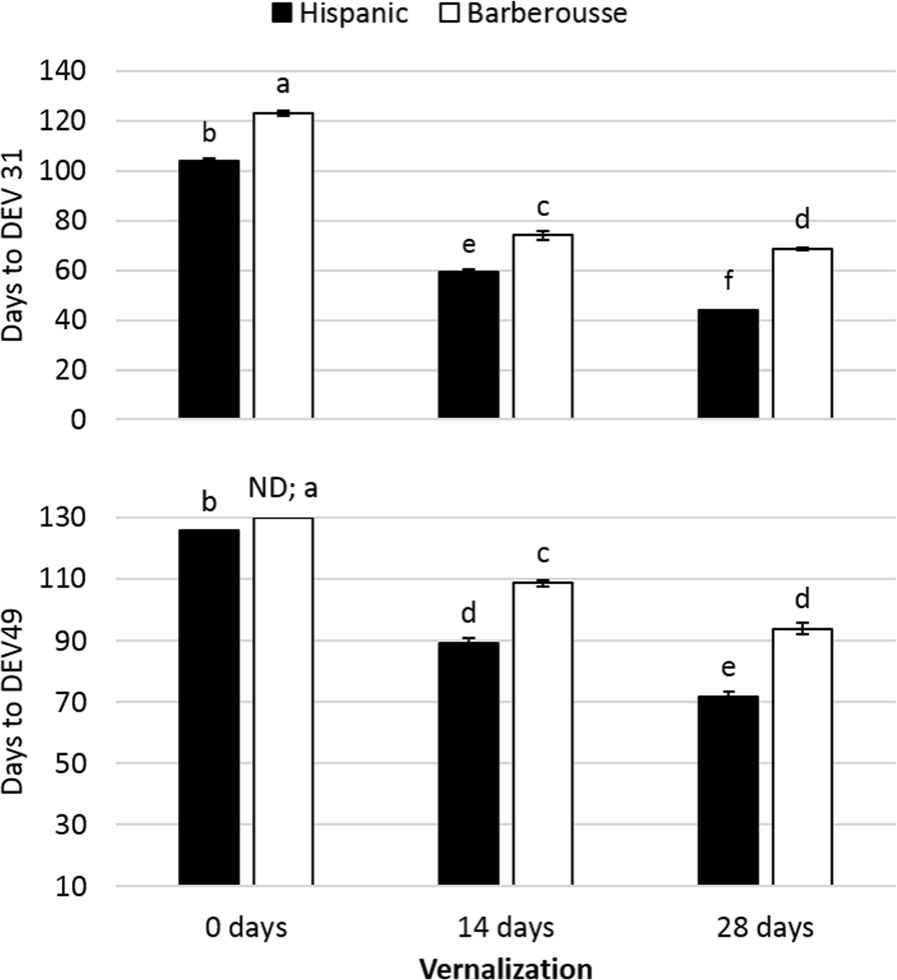
Days to appearance of first node (DEV31) and awn-tipping (DEV49) in plants grown under 12 h light, in response to different vernalization treatments. Error bars are SD. For each genotype and treatment, bars with the same letter are not significantly different at *P* < 0.05

Time to awn tipping was shortened in an inversely proportional manner to the duration of the VER treatment (Fig. 4). NV ‘Hispanic’ plants reached awn tipping (DEV49) after 126 days, whereas ‘Barberousse’ did not reach that stage during the entire duration of the experiment (136 days). Plants from both VER treatments reached DEV49 before the NV plants did. Most of this shortening occurred in the period until first node appearance (DEV31), although some additional acceleration was observed between DEV31 and DEV49.

**Fig. 5:**
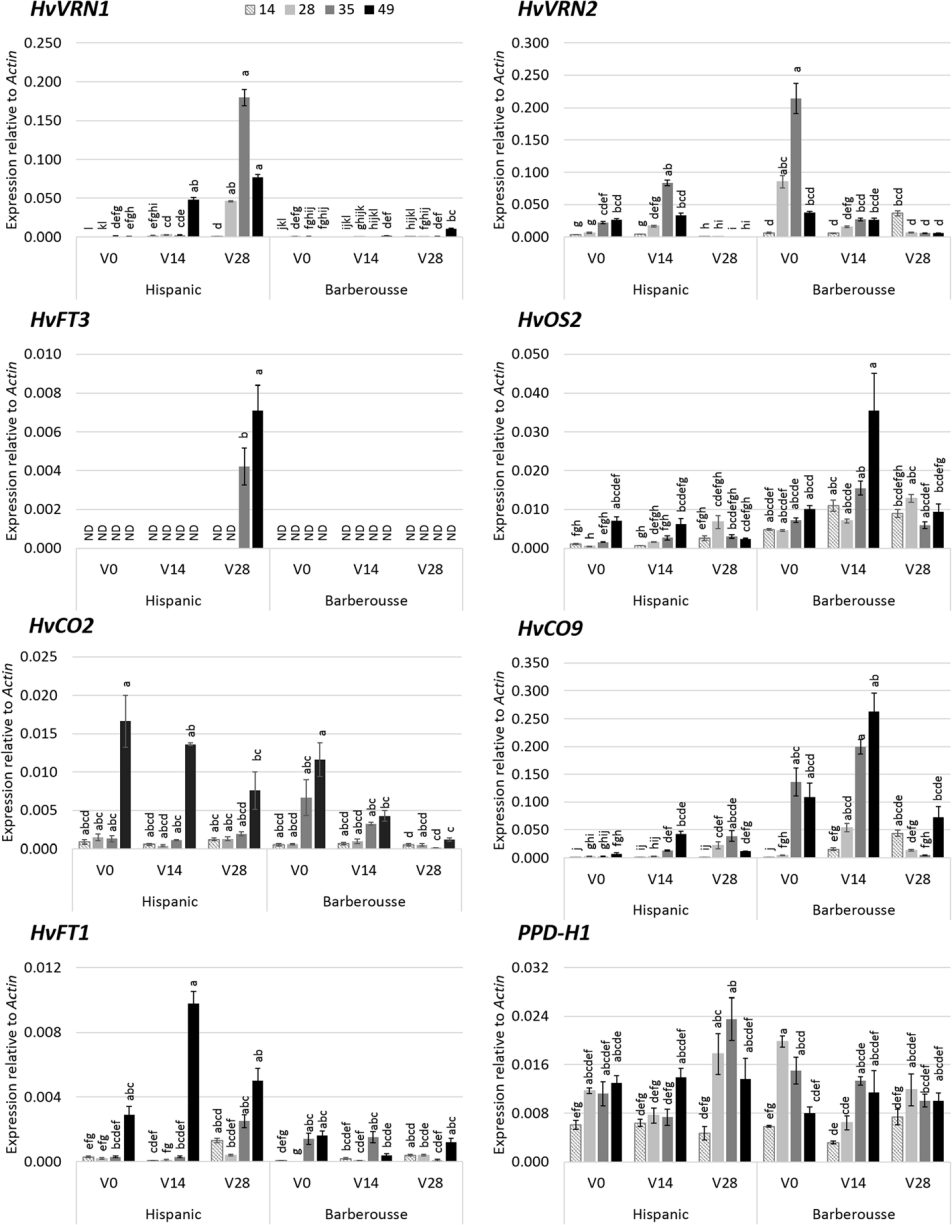
Gene expression under 12 h daylight in growth chamber. X-axis represent days of vernalization chamber. Increasing grey scale is the days after the end of the vernalization treatment when leaves were sampled (14, 28, 35 or 49 days). Mean of 3 biological replicates. Error bars are SEM. For each genotype, treatment and sampling time-point, bars with the same letter are not significantly different at *P* < 0.05 according to ANOVA that included all factors

**Fig. 6:**
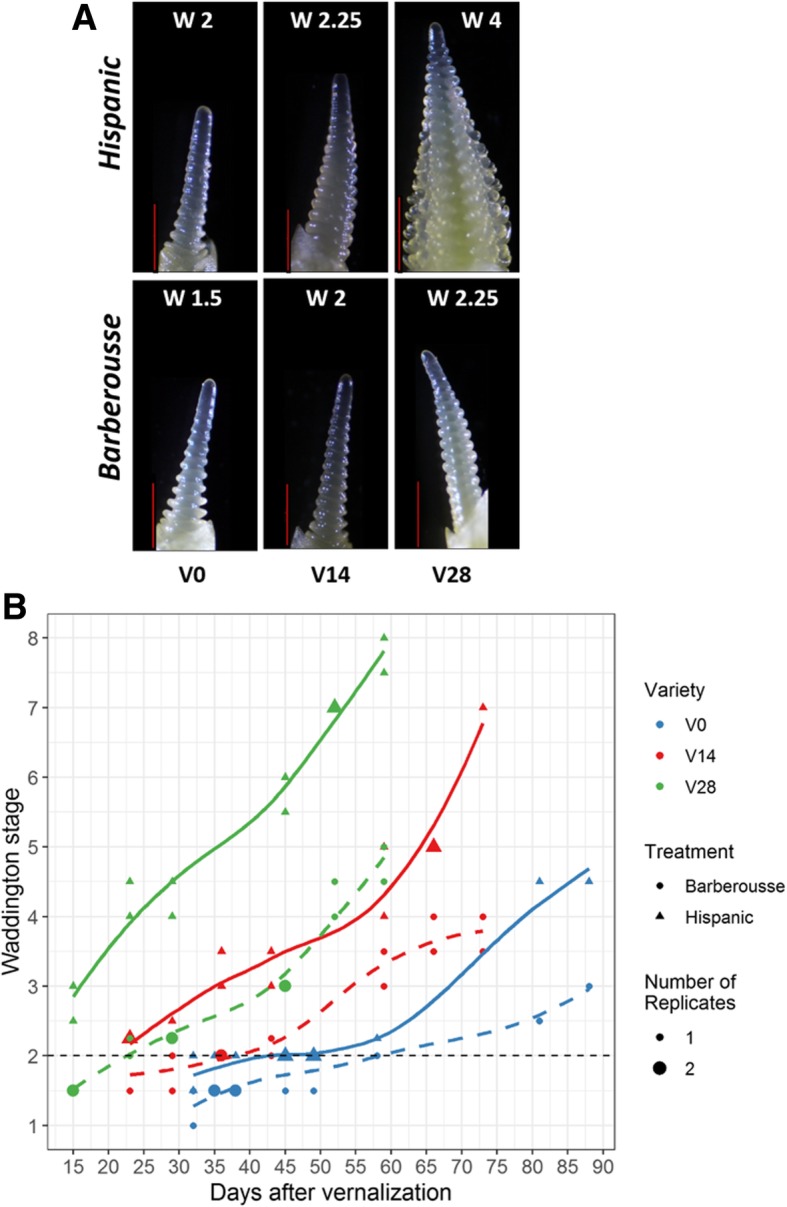
Apex dissection of plants grown under 12 h light. **a**) 4 weeks after each vernalization treatment. Red bar is 500 μm. **b**) Apex development over time after different vernalization durations. Solid lines correspond to ‘Hispanic’ and dashed lines to ‘Barberousse’. The size of each dot represent the number of apices (biological replicates) at that Waddington stage. Black dashed horizontal line marks WD2: the double ridge stage, considered as transition from vegetative to reproductive phase

Expression analysis showed higher *HvVRN1* induction the longer the VER duration in both varieties (Fig. 5, Additional file 1: Table S4). Concurrently to the larger expression of *HvVRN1, HvVRN2* was repressed, as expected. Expression of *HvCO9* and *HvOS2* was also reduced with increasing VER. These three repressors showed higher levels in ‘Barberousse’ than in ‘Hispanic’ (Fig. 5), which is in accordance with the delayed flowering of ‘Barberousse’ (Figs. 4 and 6). A similar pattern of expression of *HvCO2* and *HvFT1* was observed (*r* = 0.61). Expression of *HvCO2* was markedly higher at 49 days, with an overall trend of increase with plant age (Fig. 5). *HvFT3* transcript levels were present in ‘Hispanic’, only after plants where 28-days VER, and concurrent with a total absence of *HvVRN2*.

The increased expression levels of the flowering promoter genes and the decreased levels of the flowering repressors (Fig. 5) across treatments and genotypes agree with the patterns of development observed (Fig. 6). Four weeks after vernalization, apex transition from vegetative to reproductive stage (from W2 to W3; Fig. 6a), occurred only in ‘Hispanic’ VER 28 days plants. ‘Barberousse’ apices at all treatments, and ‘Hispanic’, VER 0 or 14 days, only reached this stage much later in time (Fig. 6b).

### Sequence polymorphisms of *HvCO2*, *HvCO9* and *HvOS2* between ‘Barberousse’ and ‘Hispanic’

**Fig. 7:**
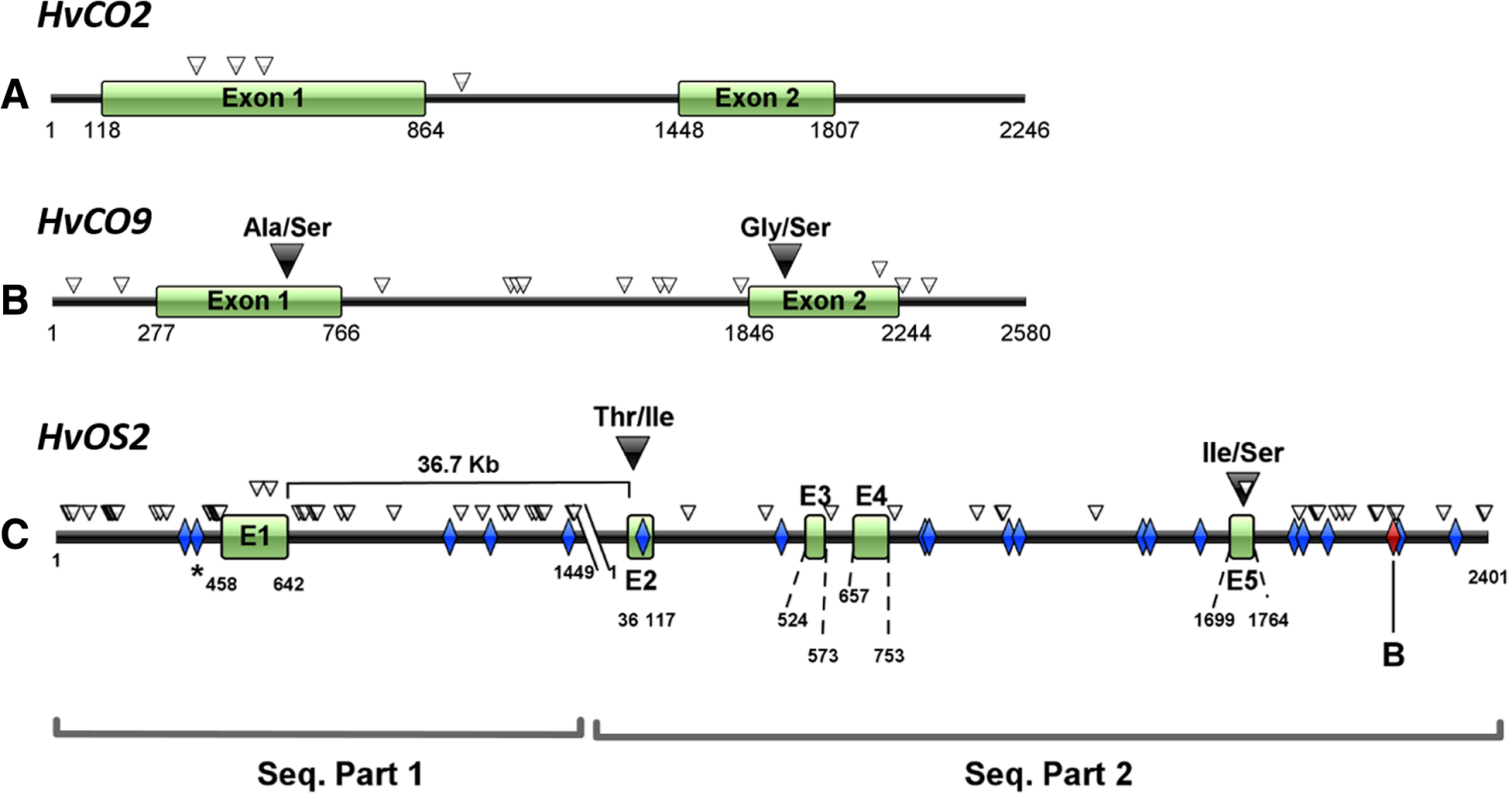
Gene sequences of **a**) *HvCO2*, **b**) *HvCO9* and **c**) *HvOS2*, and polymorphisms between ‘Barberousse’ and ‘Hispanic’. White triangles: synonymous change of aminoacid or intron variant. Black triangles: non-synonymous polymorphism. Diamonds: predicted VRN1-target sites (Deng et al. [23]). Blue diamonds: sites are conserved. Red diamonds, sites appear only in ‘Barberousse’ (B) or ‘Hispanic’ (H)

We analyzed the complete nucleotide sequences of ‘Barberousse’ and ‘Hispanic’ for the genes *HvCO2*, *HvCO9,* and partial sequences for *HvOS2,* searching for polymorphisms that may affect protein function or regulation of expression. There were coding sequence polymorphisms between the two cultivars in all three genes (Additional file 2: Table S6, Additional file 3: Table S7 and Additional file 4: Table S8). The SNPs found in *HvCO2* were synonymous (Fig. 7a) and, therefore, unlikely to be related to functional differences. Alignment of our sequences against ‘Morex’ (AF490470) and ‘Igri’ (AF490469) *HvCO2* alleles in public databases revealed two non-synonymous SNPs (T74A, A239T), but were unlikely to alter protein function (SIFT scores of 1.00 and 0.47, respectively, Additional file 1: Figure S4A).

Three SNPs were found in the coding sequence of *HvCO9* between ‘Barberousse’ and ‘Hispanic’*,* two of them non-synonymous (Fig. 7b). Both substitutions (S116A, S196G) could affect protein function (SIFT scores of 0.00 and 0.02, respectively). The peptide sequence of domain CCT was invariable in these two lines, also when compared to ‘Morex’ (AB592332) and ‘Steptoe’ (AB592331) (Additional file 1: Figure S4B). According to the SNPs found, ‘Barberousse’ was like ‘Steptoe’ and ‘Hispanic’ as ‘Morex’ (Additional file 3: Table S7).

The sequence of *HvOS2* was split in two parts. The first one comprises part of the upstream region, exon 1 and the beginning of intron 1 (~ 800 bp out of 36.7 kb). The second part contains 35 bp at the end of intron 1, and coding and non-coding regions from remaining exons 2 to 5 (Fig. 7c). Five SNPs were found within the coding sequence, with two causing amino acid substitutions (T66I and I150S). The second one, found in ‘Hispanic’ and ‘Igri’, another winter cultivar (Additional file 1: Figure S4C), could affect protein function (SIFT scores of 1.00 and 0.00, respectively). The MADS-box domain was invariable. A high number of predicted VRN1 regulatory sites where identified throughout the gene sequence. The upstream region and intron 1 showed several polymorphisms, which could affect regulation of *HvOS2*, apart from VRN1 regulatory sites (Fig. 7c, Additional file 4: Table S8).

## Discussion

The main purpose of our study was to shed light on the genes affecting development of winter barley before they receive full vernalization. This is an understudied area in barley and other cereals, and its knowledge may open new opportunities for fine-tuning the development of new cultivars to the expected winter temperatures. Vernalization and photoperiod pathways in winter cereals and *Brachypodium* are remarkably similar [1, 3, 17, 20, 38]. This proximity has allowed a direct translation of knowledge regarding genes and mechanisms between species [17, 42, 43, 47]. Therefore, any progress made in barley will be easily transferred to other crop species, like wheat. The experiments were performed under controlled conditions, carefully chosen to respond to questions that arise when barley is grown under natural conditions. The complexity found is challenging, leading to new questions but, on the other hand, brings attention to the richness of responses within winter barleys that result from the interplay of several genes.

### Expression of *HvVRN2* is upregulated beyond 12 h 30 min natural daylight in absence of vernalization

Under typical autumn sowings, winter barley is capable of responding to long photoperiods only after fulfilling a variety-specific low temperature requirement. Current accepted hypotheses indicate that *HvVRN1* is gradually induced under SD and low temperature conditions, and then represses *HvVRN2* to promote flowering [9]. In addition, that *HvVRN2* expression is triggered by LD (16 h light) and downregulated in SD (8 h light), to almost complete repression [9].

Surprisingly, we found expression of *HvVRN2,* even if at low levels, in NV plants under natural SD (sowings 1–4 in experiment 1). This finding opens the possibility that *HvVRN2* may have an effect in autumn sowings, prior to its downregulation by *HvVRN1*. Although it was not expected, some recently published experiments agree with this result. Research in *Brachypodium* found low expression of the *HvVRN2* orthologue in short days, with level of expression dependent on day-length [43]. In barley, expression of *HvVRN2* under SD, caused by overexpression of *HvCO2*, has also been reported [44]. Sampling time was not optimum for *HvCO2* in our experiments, since this gene is expressed mainly during the night [32, 33]. Accordingly, the levels of *HvCO2* expression detected in our study were low. This notwithstanding, we observed shifts in its expression, concurrent with changes in *HvVRN2* expression, which confirm their connection as hypothesized by Mulki and von Korff [44]. *HvVRN2* gene expression remained low until a surge around sowing event 5 (Fig. 2), coincident with an increase of natural daylight between 12 - 13 h (end of March), and a concurrent change of pattern of expression of *HvCO2*, responding to photoperiod cues, in unvernalized conditions. We propose that these events indicate an important shift in gene expression patterns in winter barleys, which could have an effect in plant development. In this sense, Karsai et al. [45] also found a heading date QTL, co-locating with *HvVRN2*, but only when day-length exceeded 12 h, although it is possible that the vernalization period of 42 days provided in that experiment was not enough to fulfil the needs of all those plants. This is more evidence that the role of an active *HvVRN2* allele has observable phenotypic consequences at around 12 h of day-length.

The control of these two genes has been linked to *PPD-H1*. Mulki and von Korff [44] presented evidence of a feedback loop, between *HvVRN2* and *PPD-H1*, whereas the induction of *HvCO2* by *PPD-H1*, proposed in the past [1], is currently questioned [46, 47]. A competition between VRN2 and CO2 proteins for binding to NF-Y proteins has been reported [36], which is consistent with the feedback loop described by Mulki and von Korff [44] for non-vernalized plants. *PPD-H1* shows a broad expression peak around 12 h of light in LD [32, 33]. Consequently, to reach maximum expression levels, days of 12 h or longer are required. The gradual increase of expression of *HvCO2* and *HvVRN2* with longer days observed in our study is consistent with their position downstream of *PPD-H1*. The tipping point at 12 h 30 min actually agrees with the date when natural day-length surpasses the maximum expression threshold for *PPD-H1*.

### Earliness differences between two unvernalized winter genotypes are not due to *HvVRN2* levels

The comparison of the two unvernalized winter cultivars showed a faster early development of ‘Hispanic’, as revealed by differences in pace of apex development. In both experiments ‘Hispanic’ developed or flowered always earlier than ‘Barberousse’. Differences in *HvVRN2* expression cannot be the only cause of earliness differences. This indicates the presence of additional factors affecting differentially apex development in ‘Hispanic’ and ‘Barberousse’, in the absence of vernalization.

The two cultivars differ in an unknown, but surely large, number of genes. We cannot be sure which genes are causing the differences in earliness between them. However, these differences are manifested in plants without full vernalization, and seem related to vernalization responsiveness. Therefore, it is justified to look into other genes that may act in the vernalization pathway. Currently, there is enough evidence substantiating that expression of *OS2* genes in winter cereals is suppressed by cold and could have a role in the process of vernalization. It has been proposed that *Brachypodium BdODDSOC2* “plays a role in setting the length of the vernalization requirement in a rheostatic manner, i.e. higher *ODDSOC2* transcript levels before cold result in a longer cold period needed to saturate the vernalization requirement” [20], although its specific role in the vernalization response is not clear. Across our experiments, expression of *HvOS2* was concurrent with the absence of *HvVRN1*, being lowest in plants that flowered. This coincidence was already observed in barley, wheat and *Brachypodium* [20, 21]. In addition, in the two experiments (all samplings, except one point in experiment 1), ‘Barberousse’ consistently showed higher levels of *HvOS2* transcripts than ‘Hispanic’, what agrees with the delayed development and later *HvVRN1* appearance observed in ‘Barberousse’. The predicted amino acid sequences from *HvOS2* showed polymorphisms in the coding sequence between both varieties, entailing potential change in protein function, and many other polymorphisms in non-coding regions, which could explain the expression differences. This is the first report describing sequence variation in *HvOS2*.

Recently, it was shown that the protein VRN1 binds to the promoters of *VERNALIZATION2* and *ODDSOC2* in barley [22]. Therefore, we explored the possibility that the genotypes differed in VRN1 binding sites. We identified putative VRN1-regulatory sites in *HvOS2*, and found that most of them were identical in both genotypes, leading us to exclude them as cause of dissimilar gene expression among genotypes. However, the variations in intron 1 and some VRN1-regulatory sites in 3’UTR might indicate regulatory differences among the cultivars, as found in *Arabidopsis*. Considerable natural variation in noncoding regions, affecting regulation of *FLC* (homolog of *HvOS2*) has been reported in *Arabidopsis* [48–51]. Future research will be needed to ascertain the involvement of *HvOS2* in the vernalization mechanism and the effect of the polymorphisms found in coding and non-coding sequences.

### *HvFT3* expression is not constitutive in winter cultivars, it needs induction by cold and plant development

We found differences in responsiveness to SD between the genotypes. ‘Hispanic’ developed faster than ‘Barberousse’ and flowered without vernalization. It also flowered earlier in the natural photoperiod experiment, when day-lengths increased from SD to LD (72 days in the first sowing event, without vernalization), than under SD conditions only (126 days at 12 h, Fig. 4). The two varieties differ (among others) in the presence/absence of *HvFT3.* We hypothesized that this could be a key factor differentiating their response to insufficient vernalization. This gene bears particular agronomic relevance for Mediterranean environments, as it stands at the peak of flowering time QTL and grain yield QTL x Environment peaks in several populations [28, 52–55]. A supporting role for promotion to flowering in winter cultivars, receiving less than full vernalization under field conditions, was proposed for *HvFT3* [30]. Its expression is usually reported in SD, although it is also found in LD conditions [25, 27]. In our experiments, *HvFT3* transcripts were only detected: (a) after full or partial vernalization, in early-medium development (Figs. 2 and 5), and (b) in absence of vernalization, in rather late developmental stages, and only in plants sown under shortest day-lengths (Fig. 3). We expected expression of *HvFT3*, the “short photoperiod” gene, at least in the earliest sowings in the experiment with natural photoperiods. Instead, it was effectively repressed, either by the low but always present *HvVRN2*, or by other repressors. Under constant photoperiod of 12 h, *HvFT3* was detected in ‘Hispanic’ only after four weeks VER (2 weeks were insufficient) and 5 weeks in growth chamber (Fig. 5). Thus, *HvFT3* was expressed in a winter cultivar only after some cold exposure, and increasingly with plant age. It is particularly remarkable that the expression of *HvFT3* was correlated with earlier flowering, although it was detected only after the transition from vegetative to reproductive apex had occurred (Fig. 6). This late effect on development is consistent with findings in spring wheat varieties [56], and in barley [31]. This last study evidenced that genotypes with *HvFT3* accelerated the initiation of spikelet primordia and the early reproductive development but required LD to flower.

The induction of *HvFT3* in sowing event 2 (Fig. 3), together with the progressive increase of the transcripts after 28-days VER, when *HvVRN2* is not detected, are consistent with the antagonistic role between *HvVRN2* and *HvFT3* revealed by Casao et al. [27]. *HvVRN2* absence allows induction of *HvFT3,* although it would not ensure *HvFT3* expression, hinting at the possible involvement of other repressors. In this respect, a possible relationship of *HvOS2* with *HvFT3* was suggested in the literature [57]. Future research on this possible role would shed light on the control of *HvFT3*.

*HvFT3* expression occurred in samplings coincident with that of *HvVRN1* and *HvFT1*. Parallel expression of *FT* genes has been found in grasses. Lv et al. [58] reported that developmental changes regulated by *FT1* were related to transcript levels of other *FT*-like genes, such as *FT3,* in *Brachypodium* and wheat. Under LD, these authors only found upregulation of *FT3* when *FT1* was upregulated, as in our findings with 12 h day-length. Their concurrent expression could be related to the interactions between FT1 and other FT-like proteins, including FT3, with proteins FD-like and 14–3-3, all components of the florigen activation complex (FAC), in wheat and barley [59].

## Conclusions

The results reported do not provide a full description of the dynamics of gene expression, and the conclusions derived are limited to conditions tested. Nevertheless, they open a series of questions that are worthy of further research.

The use of different sowing events, under natural increasing photoperiod corroborate that *HvVRN2* transcript levels are always present in absence of a cold-effective induction, and that the level of expression of *HvVRN2* is highly dependent on day-length. We provide evidence that the plants exhibit a shift in the pattern of expression of genes from the vernalization and photoperiod pathways, when day-length reaches around 12 h 30 min. To isolate these effects from genetic background, additional research with isogenic lines will be needed. In particular, future experiments combining sequential sowings in natural photoperiod with gradual vernalization treatments would shed light on possible effects on plant development and potential agronomic consequences of the expression shift observed, when vernalization is not complete. Further research to ascertain these possible agronomic effects with segregating populations and isogenic lines for *HvVRN2* is underway.

Other repressors appear to be acting in the process of vernalization. *HvOS2* is a suitable candidate, given the evidence accumulating in other grasses, and the genotypic differences found in our study. This hypothesis should be tested with plant materials sharing genetic background, to avoid confounding effects of other segregating genes.

*HvFT3*, a central gene for winter barley performance in Southern Europe, is not induced just by short days. In winter cultivars, it must receive additional induction through LD, and/or a cold period, to be effective in reducing time to flowering.

The photoperiod conditions of the experiments here described correspond to a wide range of late spring sowings for winter barley in the Mediterranean area. The genetic mechanisms and the environmental controls investigated in this study will be useful to define both varieties and agronomics of winter cereals best suited for current and future climate conditions.

## Methods

### Plant materials

Two winter cultivars, representative of barleys grown in Spain, with adaptation patterns likely related to differences in vernalization requirement [40, 41], were studied. ‘Barberousse’ (six-rowed, (‘Hauter’ x (‘Hatif de Grignon’ x ‘Ares’)) x ‘Ager’) is an old French cultivar developed by Ringot and registered in 1977, well adapted to the coldest areas of Spain. ‘Hispanic’ (two-rowed, ‘Mosar’ x (‘Flika’ x ‘Lada’)) is a French commercial cultivar developed by Florimond Desprez and registered in 1993, showing broad adaptation in Spain, and even acceptable agronomics in the Nile delta [41]. Both cultivars were multiplied in isolation at the EEAD-CSIC farm, collected from bagged spikes, from original seed provided by the companies. They have the same allelic combination in *HvVRN1* (winter allele), *HvVRN2*, and *PPD-H1*, but differ in *HvFT1* and *HvFT3* (*PPD-H2*, present in ‘Hispanic’, defective allele in ‘Barberousse’) [41].

### Plant growth, phenotyping and sampling

#### Experiment 1 – sowings under increasing natural photoperiod

For each variety, we used two 1 L-pots at each sowing time (standard substrate made of peat, fine sand and perlite, from a mix with 46 kg, 150 kg and 1 L, respectively). Pots were sown with 7 seeds once a week, sequentially, from Feb 11th until April 8th 2015, in a glasshouse in Zaragoza (41°43′N, 00°49′W) under natural photoperiod (Fig. 1) and controlled temperature (22 ± 1 °C day / 18 ± 1 °C night). Unless specified, plants were not vernalized (NV). Spatial homogeneity in irradiance was obtained rotating the plants each week. As vernalized control, three pots of each variety were sown on Feb 11th. They were grown during 7 days (until germination) under glasshouse conditions, and then were vernalized (VER) under short photoperiod (8 h light) and 6 ± 2 °C for 49 days. After the cold treatment, plants were transferred to the same glasshouse on April 8th, when natural photoperiod was 13 h. Duration of daylight at sowing and sampling dates was gathered from http://www.timeanddate.com/sun, taking sunrise and sunset as the times when the upper edge of the Sun’s disc touches the horizon.

For gene expression, the last expanded leaf of three 21-day-old plants (3-leaf stage) was sampled 8 h after dawn, frozen in liquid nitrogen, homogenized (Mixer Mill model MM301, Retsch) and conserved at − 80 °C until RNA isolation.

On a fixed date (19th May, day-length 15 h, 97 days after the first sowing), we took a cross-sectional sample across sowing events. The last expanded leaf of each weekly-sown plant was sampled 12 h after dawn for RNA isolation. Then, dissection of the plants (all stems of each plant) was made in order to determine the development of the apex (with naked eye, reproductive apex was equivalent to more than 3 mm).

#### Experiment 2 – growth chamber, 12 h light

Seventy-two seeds of each variety were sown in 12-well trays (650 cc) and allowed to germinate during 7 days in a growth chamber at 12 h light, 20 °C/12 h dark, 16 °C, 65% HR and light intensity of 300 μmol m^− 2^ s^− 1^ PAR. Then, the trays were divided in three groups that received the following treatments: (A) NV, (B) 14-days VER and (C) 28-days VER. Group A stayed at the growth chamber while B and C were transferred to a vernalization chamber, 8 h light/16 h night and constant temperature (6 ± 2 °C). Groups B and C were returned to the growth chamber after 14 and 28 days of cold treatment, respectively. After forty days at the growth chamber, three plants of each variety and treatment were transferred to a 1 L pots to let them grow until flowering. Development according to the Zadoks scale (first node, DEV31, and awn appearance DEV49) [60] was recorded along the experiment every 3–5 days. In addition, apex dissections were carried out at selected time points to establish the Waddington developmental stage [61]. The experiment ended 136 days after sowing.

For gene expression, the last expanded leaf of four plants was sampled 14, 28, 35 or 49 days after germination (A) or after the end of the VER treatment (B and C), 10 h into the light period (2 h before the end of the day, as in [31, 44]).

Even though a single point may not be reflective of expression at other times during the day, in all the experiments, sampling times were chosen to capture high expression of the genes involved, taking into account the period and amplitude of their circadian rhythms. *HvVRN2* expression was tested in leaf samples, taken at different times along the light period in ‘Barberousse’ plants (28 days old, and 16 h light) (Additional file 1: Figure S5), with high and comparable expression levels throughout the day.

### Vernalization response of ‘Hispanic’ and ‘Barberousse’

In the course of earlier experiments, carried out in the Phytotron of Martonvásár (Hungary), both varieties were exposed to different VER treatments (0, 15, 30 or 45 days, 5 ± 2 °C, 8 h light), and then transferred to a growth chamber with 16 h day-length, 18 °C and light intensity of 340 μmol m^− 2^ s^− 1^. Flowering date was recorded at each treatment (Additional file 1: Figure S1).

### Gene expression analysis

RNA extraction was carried out using NucleoSpin RNA Plant Kit (Macherey-Nagel) following manufacturer instructions. Total RNA (1 μg) was employed for cDNA synthesis using SuperScript III Reverse Transcriptase (Invitrogen) and oligo (dT)_20_ primer (Invitrogen). Real-time PCR quantification (ABI 7500, Applied Biosystems) was performed for samples from each time point from NV plants and for VER plants as control treatment. Three biological replicates and two technical replicates were performed per sample and pair of primers (*HvVRN1*, *HvVRN2*, *PPD-H1*, *HvCO2*, *HvCO9*, *HvOS2, HvFT1*, and *HvFT3*). Primer sequences and conditions are specified in Additional file 1: Table S5. Expression levels were normalized to *Actin* expression, taking into account primer efficiencies.

### Gene sequencing

Polymorphisms in *HvCO2*, *HvCO9* and *HvOS2* were identified by sequencing genomic DNA PCR-amplified overlapping fragments. Primers were designed to amplify each gene (Additional file 1: Table S5). The resulting sequences have been deposited at the European Nucleotide Archive as part of project PRJEB27962. BLASTN sequence comparisons [62] were carried out against the barley Morex reference genome [63], and Morex, Barke and Bowman whole genome barley sequences [64] at the IPK (http://webblast.ipk-gatersleben.de/barley_ibsc/) web server. Sequence comparisons against NCBI nucleotide database, cv. Haruna Nijo [65] and cv. Zangqing320 genomic sequences [66] were also performed. Sequence alignments were carried out in MEGA-X v.10.0.4 [67]. Predicted protein alignments were carried out in ClustalW [68]. Protein domains were annotated according to Cockram et al. [37], Greenup et al. [21] and Prosite v20.79 (http://prosite.expasy.org/). The online tool SIFT (http://sift.bii.a-star.edu.sg) was used to predict the likely impact of amino acid substitutions on protein function, using as reference Morex [63]. Scores below 0.05 are predicted to affect protein function. Putative VRN1 regulatory elements were predicted by scanning a motif compiled from ChIP-seq peaks reported in Deng et al. [23] and annotated in http://floresta.eead.csic.es/footprintdb/index.php?motif=VRN1&db=EEADannot [69]. Briefly, upstream sequences of target barley genes were retrieved from the RSAT plant mirror (http://plants.rsat.eu, [70]) and *matrix-scan-quick* used to scan the motif using a genomic Markov model of order 2 (upstream-noorf_Hordeum_vulgare.IBSCv2.37). Only sites with weight > = 3.7 were considered.

### Statistical analysis

Statistical analyses were carried out with R software [71]. For gene expression results, the mean of two technical replications of ∆Ct (Ct actin – Ct target) was used as unit. Analyses of variance for phenotypes or gene expression data were performed considering all factors (genotype, sampling time, vernalization treatment) as fixed. Multiple comparisons were obtained by Fisher’s protected Least Significant Differences (LSD) with the R package ‘agricolae’ [72]. Pearson correlations were carried out with ‘cor’ function.

## Additional files

Additional file 1: Supplementary data. Tables S1-S5 and Figure S1-S5. (PDF 1468 kb)

Additional file 2: **Table S6.** *HvCO2* polymorphisms. A) Information of the sequences obtained for the gene *HvCO2* var. ‘Barberousse’ and var. ‘Hispanic’. B) Polymorphisms found for *HvCO2* sequences. C) Alignments of *HvCO2* gene sequences. D) Alignments of HvCO2 predicted protein sequences. (XLSX 89 kb)

Additional file 3: **Table S7.** *HvCO9* polymorphisms. A) Information of the sequences obtained for the gene *HvCO9* var. ‘Barberousse’ and var. ‘Hispanic’. B) Polymorphisms found for *HvCO9* sequences. C) Alignments of *HvCO9* gene sequences. D) Alignments of HvCO9 predicted protein sequences. (XLSX 106 kb)

Additional file 4: **Table S8.** *HvOS2* polymorphisms. A) Information of the sequences obtained for the gene *HvOS2* var. ‘Barberousse’ and var. ‘Hispanic’. B) Polymorphisms found for *HvOS2* CDS sequences. C-F) Alignments of *HvOS2* sequences - exon 1 (C), exons 2–5 (D), CDS (E), predicted protein (F). G-H) Predicted VRN1 regulatory sites in *HvOS2* exon 1 (G) and exons 2–5 (H), in var. ‘Barberousse’ and var. ‘Hispanic’. (XLSX 161 kb)

## Abbreviations

*AP1*: *APETALA1*
*CO*: *CONSTANS*
*FLC*: *FLOWERING LOCUS C*
*FT*: *FLOWERING LOCUS T*
LD: Long day
NV: Non-vernalized
SD: Short day
VER: vernalized
*ZCCT-H*: zinc-finger CCT-domain *Hordeum* containing genes

## References

[1] Campoli C, von Korff M (2014). Genetic control of reproductive development in temperate cereals. Adv Bot Res.

[2] Digel B, Pankin A, von Korff M (2015). Global transcriptome profiling of developing leaf and shoot apices reveals distinct genetic and environmental control of floral transition and inflorescence development in barley. Plant Cell.

[3] Trevaskis B (2010). The central role of the *VERNALIZATION1* gene in the VERNALIZATION response of cereals. Funct Plant Biol.

[4] Tao F, Rötter RP, Palosuo T, Díaz-Ambrona CGH, Mínguez MI, Semenov MA (2017). Designing future barley ideotypes using a crop model ensemble. Eur J Agron.

[5] Song YH, Kubota A, Kwon MS, Covington MF, Lee N, Taagen ER (2018). Molecular basis of flowering under natural long-day conditions in Arabidopsis. Nat Plants.

[6] Distelfeld A, Li C, Dubcovsky J (2009). Regulation of flowering in temperate cereals. Curr Opin Plant Biol.

[7] Yan L, Loukoianov A, Blechl A, Tranquilli G, Ramakrishna W, SanMiguel P (2004). The wheat *VRN2* gene is a flowering repressor down-regulated by vernalization. Science..

[8] Karsai I, Szucs P, Mészáros K, Filichkina T, Hayes PM, Skinner JS (2005). The *Vrn-H2* locus is a major determinant of flowering time in a facultative x winter growth habit barley (*Hordeum vulgare* L.) mapping population. Theor Appl Genet.

[9] Trevaskis B, Hemming MN, Peacock WJ, Dennis ES (2006). *HvVRN2* responds to daylength, whereas *HvVRN1* is regulated by vernalization and developmental status. Plant Physiol.

[10] Danyluk J, Kane NA, Breton G, Limin AE, Fowler DB, Sarhan F (2003). TaVRT-1 , a putative transcription factor associated with vegetative to reproductive transition in cereals. Plant Physiol.

[11] Trevaskis B, Bagnall DJ, Ellis MH, Peacock WJ, Dennis ES (2003). MADS box genes control vernalization-induced flowering in cereals. Proc Natl Acad Sci.

[12] Yan L, Loukoianov A, Tranquilli G, Helguera M, Fahima T, Dubcovsky J (2003). Positional cloning of the wheat vernalization gene *VRN1*. Proc Natl Acad Sci.

[13] Hemming MN, Fieg S, James Peacock W, Dennis ES, Trevaskis B (2009). Regions associated with repression of the barley (*Hordeum vulgare*) *VERNALIZATION1* gene are not required for cold induction. Mol Gen Genomics.

[14] von Zitzewitz J, Szucs P, Dubcovsky J, Yan L, Francia E, Pecchioni N (2005). Molecular and structural characterization of barley vernalization genes. Plant Mol Biol.

[15] Sasani S, Hemming MN, Oliver SN, Greenup A, Tavakkol-Afshari R, Mahfoozi S (2009). The influence of vernalization and daylength on expression of flowering-time genes in the shoot apex and leaves of barley (*Hordeum vulgare*). J Exp Bot.

[16] Oliver SN, Finnegan EJ, Dennis ES, Peacock WJ, Trevaskis B (2009). Vernalization-induced flowering in cereals is associated with changes in histone methylation at the *VERNALIZATION1* gene. Proc Natl Acad Sci.

[17] Bouché F, Woods D, Amasino RM (2017). Winter memory throughout the plant kingdom: different paths to flowering. Plant Physiol.

[18] Rizza F, Karsai I, Morcia C, Badeck F-W, Terzi V, Pagani D (2016). Association between the allele compositions of major plant developmental genes and frost tolerance in barley (*Hordeum vulgare* L.) germplasm of different origin. Mol Breed.

[19] Chen A, Dubcovsky J (2012). Wheat TILLING mutants show that the vernalization gene *VRN1* down-regulates the flowering repressor *VRN2* in leaves but is not essential for flowering. PLoS Genet.

[20] Sharma N, Ruelens P, Dhauw M, Maggen T, Dochy N, Torfs S (2017). A flowering time locus C homolog is a vernalization-regulated repressor in *Brachypodium* and is cold-regulated in wheat. Plant Physiol.

[21] Greenup AG, Sasani S, Oliver SN, Talbot MJ, Dennis ES, Hemming MN (2010). *ODDSOC2* is a MADS box floral repressor that is down-regulated by vernalization in temperate cereals. Plant Physiol.

[22] Ruelens P, de Maagd RA, Proost S, Theißen G, Geuten K, Kaufmann K (2013). FLOWERING LOCUS C in monocots and the tandem origin of angiosperm-specific MADS-box genes. Nat Commun.

[23] Deng W, Casao MC, Wang P, Sato K, Hayes PM, Finnegan EJ (2015). Direct links between the vernalization response and other key traits of cereal crops. Nat Commun.

[24] Faure S, Higgins J, Turner A, Laurie DA (2007). The *FLOWERING LOCUS T*-like gene family in barley (*Hordeum vulgare*). Genetics..

[25] Kikuchi R, Kawahigashi H, Ando T, Tonooka T, Handa H (2009). Molecular and functional characterization of PEBP genes in barley reveal the diversification of their roles in flowering. Plant Physiol.

[26] Laurie DA, Pratchett N, Snape JW, Bezant JH (1995). RFLP mapping of five major genes and eight quantitative trait loci controlling flowering time in a winter × spring barley (*Hordeum vulgare* L.) cross. Genome..

[27] Casao MC, Igartua E, Karsai I, Lasa JM, Gracia MP, Casas AM (2011). Expression analysis of vernalization and day-length response genes in barley (*Hordeum vulgare* L.) indicates that *VRNH2* is a repressor of *PPDH2* (*HvFT3*) under long days. J Exp Bot.

[28] Cuesta-Marcos A, Igartua E, Ciudad FJ, Codesal P, Russell JR, Molina-Cano JL (2008). Heading date QTL in a spring × winter barley cross evaluated in Mediterranean environments. Mol Breed.

[29] Borràs-Gelonch G, Denti M, Thomas WTB, Romagosa I (2012). Genetic control of pre-heading phases in the Steptoe x Morex barley population under different conditions of photoperiod and temperature. Euphytica..

[30] Casao MC, Karsai I, Igartua E, Gracia MP, Veisz O, Casas AM (2011). Adaptation of barley to mild winters: a role for *PPDH2*. BMC Plant Biol.

[31] Mulki MA, Bi X, von Korff M (2018). FLOWERING LOCUS T3 controls spikelet initiation but not floral development. Plant Physiol.

[32] Campoli C, Shtaya M, Davis SJ, von Korff M (2012). Expression conservation within the circadian clock of a monocot: natural variation at barley *Ppd-H1* affects circadian expression of flowering time genes, but not clock orthologs. BMC Plant Biol.

[33] Turner A, Beales J, Faure S, Dunford RP, Laurie DA (2005). The pseudo-response regulator *Ppd-H1* provides adaptation to photoperiod in barley. Science..

[34] Griffiths S, Dunford RP, Coupland G, Laurie DA (2003). The evolution of *CONSTANS*-like gene families in barley, rice, and Arabidopsis. Plant Physiol.

[35] Nemoto Y, Kisaka M, Fuse T, Yano M, Ogihara Y (2003). Characterization and functional analysis of three wheat genes with homology to the *CONSTANS* flowering time gene in transgenic rice. Plant J.

[36] Li C, Distelfeld A, Comis A, Dubcovsky J (2011). Wheat flowering repressor VRN2 and promoter CO2 compete for interactions with NUCLEAR FACTOR-Y complexes. Plant J.

[37] Cockram J, Thiel T, Steuernagel B, Stein N, Taudien S, Bailey PC (2012). Genome dynamics explain the evolution of flowering time CCT domain gene families in the Poaceae. PLoS One.

[38] Higgins JA, Bailey PC, Laurie DA (2010). Comparative genomics of flowering time pathways using *Brachypodium distachyon* as a model for the temperate grasses. PLoS One.

[39] Kikuchi R, Kawahigashi H, Oshima M, Ando T, Handa H (2012). The differential expression of *HvCO9*, a member of the *CONSTANS*-like gene family, contributes to the control of flowering under short-day conditions in barley. J Exp Bot.

[40] Igartua E, Casas AM, Ciudad F, Montoya JL, Romagosa I (1999). RFLP markers associated with major genes controlling heading date evaluated in a barley germ plasm pool. Heredity.

[41] Mansour E, Moustafa ESA, Qabil N, Abdelsalam A, Wafa HA, El Kenawy A, Casas AM, Igartua E (2018). Assessing different barley growth habits under Egyptian conditions for enhancing resilience to climate change. Field Crops Res.

[42] Turner AS, Faure S, Zhang Y, Laurie DA (2013). The effect of day-neutral mutations in barley and wheat on the interaction between photoperiod and vernalization. Theor Appl Genet.

[43] Woods D, Dong Y, Bouche F, Bednarek R, Rowe M, Ream T, Amasino R (2019). A florigen paralog is required for short-day vernalization in a pooid grass. eLife.

[44] Mulki MA, von Korff M (2016). *CONSTANS* controls floral repression by up-regulating *VERNALIZATION2* ( *VRN-H2* ) in barley. Plant Physiol.

[45] Karsai I, Meszaros K, Szucs P, Hayes PM, Lang L, Bedo Z (2006). The influence of photoperiod on the *Vrn-H2* locus (4H) which is a major determinant of plant development and reproductive fitness traits in a facultative x winter barley (*Hordeum vulgare* L.) mapping population. Plant Breed.

[46] Chen A, Li C, Hu W, Lau MY, Lin H, Rockwell NC (2014). PHYTOCHROME C plays a major role in the acceleration of wheat flowering under long-day photoperiod. Proc Natl Acad Sci U S A.

[47] Song YH, Shim JS, Kinmonth-Schultz HA, Imaizumi T (2015). Photoperiodic flowering: time measurement mechanisms in leaves. Annu Rev Plant Biol.

[48] Gazzani S, Gendall AR, Lister C, Dean C (2003). Analysis of the molecular basis of flowering time variation in Arabidopsis accessions. Plant Physiol.

[49] Michaels SD, He Y, Scortecci KC, Amasino R (2003). Attenuation of FLOWERING LOCUS C activity as a mechanism for the evolution of summer-annual flowering behavior in *Arabidopsis*. Proc Natl Acad Sci U S A.

[50] Li P, Filiault D, Box MS, Kerdaffrec E, Van Oosterhout C, Wilczek AM (2014). Multiple FLC haplotypes defined by independent cis -regulatory variation underpin life history diversity in *Arabidopsis thaliana*. Genes Dev.

[51] Whittaker C, Dean C (2017). The *FLC* locus: a platform for discoveries in epigenetics and adaptation. Annu Rev Cell Dev Biol.

[52] Cuesta-Marcos A, Casas AM, Hayes PM, Gracia MP, Lasa JM, Ciudad F (2009). Yield QTL affected by heading date in Mediterranean grown barley. Plant Breed.

[53] Karsai I, Szucs P, Koszegi B, Hayes PM, Casas AM, Bedo Z (2008). Effects of photo and thermo cycles on flowering time in barley: a genetical phenomics approach. J Exp Bot.

[54] Francia E, Tondelli A, Rizza F, Badeck FW, Li Destri Nicosia O, Akar T (2011). Determinants of barley grain yield in a wide range of Mediterranean environments. F Crop Res.

[55] Tondelli A, Francia E, Visioni A, Comadran J, Mastrangelo AM, Akar T (2014). QTLs for barley yield adaptation to Mediterranean environments in the “Nure” × “Tremois” biparental population. Euphytica..

[56] Halliwell J, Borrill P, Gordon A, Kowalczyk R, Pagano ML, Saccomanno B (2016). Systematic investigation of *FLOWERING LOCUS T*-like poaceae gene families identifies the short-day expressed flowering pathway gene, *TaFT3* in wheat (*Triticum aestivum* L.). Front Plant Sci.

[57] Cuesta-Marcos A, Muñoz-Amatrián M, Filichkin T, Karsai I, Trevaskis B, Yasuda S (2015). The relationships between development and low temperature tolerance in barley near isogenic lines differing for flowering behavior. Plant Cell Physiol.

[58] Lv B, Nitcher R, Han X, Wang S, Ni F, Li K (2014). Characterization of *Flowering Locus T1* (*FT1*) gene in *Brachypodium* and wheat. PLoS One.

[59] Li C, Lin H, Dubcovsky J (2015). Factorial combinations of protein interactions generate a multiplicity of florigen activation complexes in wheat and barley. Plant J.

[60] Zadoks JC, Chang TT, Konzak CF (1974). A decimal code for the growth stages of cereals. Weed Res.

[61] Waddington SR, Cartwright PM (1983). A quantitative scale of spike initial and pistil development in barley and wheat. Ann Bot.

[62] Altschul SF, Gish W, Miller W, Myers EW, Lipman DJ (1990). Basic local alignment search tool. J Mol Biol.

[63] Mascher M, Gundlach H, Himmelbach A, Beier S, Twardziok SO, Wicker T (2017). A chromosome conformation capture ordered sequence of the barley genome. Nature..

[64] Mayer KFX, Waugh R, Langridge P, Close TJ, Wise RP, Graner A (2012). A physical, genetic and functional sequence assembly of the barley genome. Nature..

[65] Sato K, Tanaka T, Shigenobu S, Motoi Y, Wu J, Itoh T (2016). Improvement of barley genome annotations by deciphering the Haruna Nijo genome. DNA Res.

[66] Dai F, Wang X, Zhang XQ, Chen Z, Nevo E, Jin G (2018). Assembly and analysis of a qingke reference genome demonstrate its close genetic relation to modern cultivated barley. Plant Biotechnol J.

[67] Kumar S, Stecher G, Li M, Knyaz C, Tamura K (2018). MEGA X: Molecular evolutionary genetics analysis across computing platforms. Mol Biol Evol.

[68] Larkin MA, Blackshields G, Brown NP, Chenna R, McGettigan PA, McWilliam H (2007). Clustal W and Clustal X version 2.0. Bioinformatics..

[69] Sebastian A, Contreras-Moreira B (2014). footprintDB: a database of transcription factors with annotated cis elements and binding interfaces. Bioinformatics.

[70] Nguyen NTT, Contreras-Moreira B, Castro-Mondragon JA, Santana-Garcia W, Ossio R, Robles-Espinoza CD, Bahin M (2018). RSAT 2018: regulatory sequence analysis tools 20th anniversary. Nucleic Acids Res.

[71] R Core Team. R: A language and environment for statistical computing. 2017. https://www.r-project.org/. Accessed 21 Mar 2019.

[72] de Mendiburu F. agricolae: Statistical Procedures for Agricultural Research. 2016. https://cran.r-project.org/package=agricolae. Accessed 21 Mar 2019.

