# Supplementary material for: Fine-tuning of the flowering time control in winter barley: the importance of HvOS2 and HvVRN2 in non-inductive conditions
Source: BMC Plant Biol. 2019 Mar 25;19:113. doi: 10.1186/s12870-019-1727-9 (PMC6434887; doi:10.1186/s12870-019-1727-9)
Supplement: Supplementary file 1 — Supplementary data. Tables S1-S5 and Figure S1-S5. (PDF 1468 kb) [file 12870_2019_1727_MOESM1_ESM.pdf]

Supplementary data

Fine-tuning of the flowering time control in winter barley: the importance of *HvOS2* and *HvVRN2* in non-inductive conditions

A Monteagudo, E Igartua, B Contreras-Moreira, MP Gracia, J Ramos, I Karsai, AM Casas

Supplementary data

Table S1. Analysis of variance for the ΔCt corresponding to the expression of genes of 'Hispanic' and 'Barberousse' in 3-week-old plants…

Table S2. ANOVA of *HvVRN2* and *HvCO2* expression in 21 days-old plants of two varieties grown in absence of vernalization under natural photoperiods.

Table S3. Analysis of variance for the ΔCt corresponding to the expression of genes of 'Hispanic' and 'Barberousse' plants grown under natural photoperiods without vernalization, and sampled on a set date, with 15 h light…

Table S4. Analysis of variance for the ΔCt corresponding to the expression of genes of 'Hispanic' and 'Barberousse' in plants grown under 12 h light, with different vernalization treatments…

Table S5. Sequences of primers used in this work.

Figure S1. Flowering date under different vernalization treatments in the preliminary experiment…

Figure S2. Gene expression in 2-week-old plants sown under natural and increasing photoperiods (without vernalization and control)…

Figure S3. Percentage of reproductive apices with respect to the total (vegetative and reproductive) after 100 days of the experiment…

Figure S4. *HvVRN2* diurnal expression in 'Barberousse' unvernalized plants grown under LD (16 h light)…

Figure S5. Alignment of HvCO2, HvCO9 and HvOS2 proteins.

Table S1. Analysis of variance for the ΔCt corresponding to the expression of genes of 'Hispanic' and 'Barberousse' in 3-week-old plants under natural photoperiods without vernalization. Between 3 and 4 biological replicates per gene were used.

	HvVRN1			*HvVRN2*			*HvCO2*			*PPD-H1*			*HvOS2*			*HvCO9*		
Source of variation	df	ms		df	ms		df	ms		df	ms		df	ms		df	ms	
Variety	1	1.68	ns	1	25.18	**	1	0.62	ns	1	0.78	ns	1	30.94	***	1	5.85	ns
Sowing event	8	15.15	ns	8	15.92	***	8	16.21	***	8	1.18	*	8	2.24	*	8	149.06	***
Variety * Sowing event	8	8.75	ns	8	3.05	ns	8	2.77	ns	8	1.15	*	8	1.38	ns	8	31.37	ns
Residuals	34	9.30		34	2.05		35	2.20		51	0.43		35	0.81		35	90.20	

*, **, *** significant effects at $P < 0.05$, < 0.01, < 0.001

Table S2. ANOVA of A) *HvVRN2* and B) *HvCO2* expression in 21 days-old plants of two varieties grown in absence of vernalization under natural photoperiods. Plants were sown sequentially from Feb 11th to Apr 8th (sowing events 1-9). For this analysis, the nine sowing events were grouped as 1-4 (shorter photoperiod) and 5-9 (longer photoperiod), with the threshold at 12h 30m as the splitting point. This subfactor was named "Group". We subdivided the differences between sowing events and the interaction between sowing events and variety in two components: the variance due to the differences between the two groups and the variance due to differences between sowing events within groups.

**A) *HvVRN2*** Source of variation	Degrees of freedom	Sum of squares	Mean squares	F value	Pr(>F)	
Variety	1	25.18	25.18	12.28	0.0013	**
Sowing event (Sowing)	8	127.36	15.92	7.77	7.17E-06	***
Group	1	99.36	99.36	48.47	5.00E-08	***
Within group	7	28.00	4.00	1.95	0.0916	
Variety * Sowing	8	24.43	3.05	1.49	0.1976	
Variety * Group	1	4.93	4.93	2.40	0.1303	
Variety * within group	7	19.50	2.79	1.36	0.2542	
Residuals	34	69.70	2.05			

*, **, *** significant effects at P < 0.05, < 0.01, < 0.001

**B) *HvCO2*** Source of variation	Degrees of freedom	Sum of squares	Mean squares	F value	**Pr(>F)**	
Variety	1	0.62	0.62	0.28	0.599	
Sowing event (Sowing)	8	129.64	16.21	7.35	1.07E-05	***
Group	1	17.74	17.74	8.05	0.00752	**
Within group	7	111.90	15.99	7.25	2.23e-05	***
Variety * Sowing	8	22.19	2.77	1.26	0.296	
Variety * Group	1	0.25	0.25	0.12	0.736	
Variety * within group	7	21.93	3.13	1.42	0.228	
Residuals	35	77.14	2.20			

*, **, *** significant effects at P < 0.05, < 0.01, < 0.001

Table S3. Analysis of variance for the ΔCt corresponding to the expression of genes of 'Hispanic' and 'Barberousse' plants grown under natural photoperiods without vernalization, and sampled on a set date, with 15 h light. Between 3 and 4 biological replicates per gene were used.

	HvVRN1			*HvVRN2*			*HvCO2*			*PPD-H1*			*HvOS2*			*HvCO9*			*HvFT1*		
Source of variation	df	ms		df	ms		df	ms		df	ms		df	ms		df	ms		df	ms	
Variety	1	179.0	*	1	33.92	**	1	23.03	*	1	1.99	ns	1	103.11	***	1	3.73	ns	1	176.9	***
Sowing event	6	213.5	***	6	2.87	ns	6	2.34	ns	6	3.82	***	6	36.94	***	6	10.51	ns	6	93.3	***
Variety * Sowing event	6	119.7	**	6	2.55	ns	6	8.44	ns	6	1.54	*	6	10.2	ns	6	13.88	ns	6	13.3	ns
Residuals	40	28.1		40	2.92		26	4.31		26	0.54		29	5.93		33	7.04		30	10.9	

*, **, *** significant effects at P < 0.05, < 0.01, < 0.001

Table S4. Analysis of variance for the ΔCt corresponding to the expression of genes of 'Hispanic' and 'Barberousse' in plants grown under 12 h light, with different vernalization treatments (0, 14 and 28 days). Between 3 and 4 biological replicates per gene were used.

	HvVRN1			*HvVRN2*			*HvCO2*			*PPD-H1*			*HvOS2*			*HvCO9*			*HvFT1*		
Source of variation	df	ms		df	ms		df	ms		df	ms		df	ms		df	ms		df	ms	
Variety	1	222.2	***	1	116.5	***	1	4.8	ns	1	0.44	ns	1	102.8	***	1	139.4	***	1	6.2	ns
Vernalization	2	181.5	***	2	134.3	***	2	52.6	**	2	1.72	**	2	2.1	ns	2	26.3	***	2	53.4	**
Age	3	61.3	***	3	7.9	**	3	40.0	*	3	4.55	***	3	5.4	ns	3	71.6	***	3	140.2	***
Variety * Vernalization	2	88.6	***	2	64.6	***	2	9.6	ns	2	0.69	ns	2	7.2	ns	2	17.9	**	2	7.8	ns
Variety * Age	3	3.4	ns	3	1.0	ns	3	10.1	ns	3	0.21	ns	3	1.0	ns	3	1.7	ns	3	10.3	ns
Vernalization * Age	6	5.9	ns	6	12.4	***	6	6.5	ns	6	0.88	ns	6	3.2	ns	6	9.7	**	6	18.6	ns
Variety * Ver * Age	6	11.1	**	6	2.4	ns	6	10.8	ns	6	1.05	ns	6	1.7	ns	6	18.5	***	6	16.7	ns
Residuals	48	2.6		48	1.4		47	14.1		48	0.53		48	2.8		48	2.9		49	10.4	

*, **, *** significant effects at $P < 0.05$, < 0.01, < 0.001

Table S5. Primer sequences for gene expression assay and sequencing.

Gene expression

**Gene**	**Primer sequence (5'-3')**	**Reference**
HvCO2 [b]	Forward: CATCACTTGTGACCCAAGACC<br>Reverse: CTATAGTTCCATAATTGCTCC	[1]
HvCO9 [a]	Forward: AAGCTGATGCGGTACAAAGAGA<br>Reverse: GAACCACCCGAGGTCGAG	[2]
HvFT1 [a]	Forward: ATCTCCACTGGTTGGTGACAGA<br>Reverse: TTGTAGAGCTCGGCAAAGTCC	[3]
HvFT3 [b]	Forward: GGTTGTGGCTCATGTTATGC<br>Reverse: CTACTCCCCTTGAGAACTTTC	Forward: [4]; Reverse: [5]
HvOS2 [a]	Forward: CAATGCTGATGACTCAGATGCT<br>Reverse: CGCTATTTCGTTGCGCCAAT	[6]
Ppd-H1[b]	Forward: CAAATCAAAGAGCGGCGATC<br>Reverse: TCTGACTTGGGATGGTTCACA	[7]
HvVRN1[a]	Forward: TATGAGCGCTACTCTTATGC<br>Reverse: TGAAGCTCAGAAATGGATTCG	[8]
HvVRN2 [a]	Forward: GAGCCACCATCGTGCCATTC<br>Reverse: GCCGCTTCTTCCTCTTCTC	[8]
Actin	Forward: GCCGTGCTTTCCCTCTATG<br>Reverse: GCTTCTCCTTGATGTCCCTTA	[8]

Sequencing

**Gene**	**Primer sequence (5'-3')**	**Length (bp)**	**Region**
HvCO2	**Morex_contig_6805**		
CO2.1F	TTTTCGCTCACTGGATTCCAC	983	5' UTR, exon 1
CO2.2R	GCCTTGAAGTGGTACGAACTC		
CO2.7F	GCAAGGGAGCAATTATGGAA	988	Exon1, intron, exon 2
CO2.8R	GTGGTGACAGCATGTGGTTC		
CO2.5F	TAGTACCGGACAACACCAGAC	782	Exon 2, 3' UTR
CO2.6R	TCCCCAAGAAGTGTGTATCCA		
HvCO9	**Morex_contig_67944**		
CO9.1F	ACGTAAGGAACCCTCCCATC	996	5'UTR, exon 1

CO9.2R	TAACAATTTGCACCACACGC		
CO9.3F	AGTTCCAGTTCTTCGGGCAG	783	Exon 1, intron
CO9.4R	ATTACTAGTGTGGGCCGAGG		
CO9.5F	CAAATATTTCTGCGTGTGGTGC	949	Intron
CO9.6R	CGTTTCTCTCCCGCAATAAGG		
CO9.7F	TTCGAAATGCCATGCTCTTCC	910	Exon 2, 3'UTR
CO9.8R	CCGCCCCACCTCAATTTATTT		
HvOS2	**HORVU3Hr1G095240**		
OS2.1F	TTTTTCACAGCGTGGATAAGG	936	5'UTR, exon 1, intron
OS2.2R	TTGGCCGTGACAATAATAAGC		
OS2.3F	CCGGTAAATCAAGGCTGCTC	829	Intron 1, partial
OS2.4R	GCACAACAAACTCTCGGTGA		
OS2.5F	ATTTCCAGCAGAGCCTAAAGC	918	Exon2-exon 4
OS2.6R	TGAAAATGGCCAAAAACAGAGC		
OS2.7F	ACTTCCTGGTAGCCCTTGAG	854	Exon 4, intron 4
OS2.8R	ACAACAGAGCCAACTTGTCG		
OS2.9F	CTTAGTTGCTGCAGTCTCACTC	877	Intron 4, exon 5
OS2.10R	TACCAAATGCATGCACATCACA		
OS2.11F	AGAGCCTGGCATGAGAGTTC	855	Exon 5, 3'UTR
OS2.12R	CCCAGGGAAGACACTTGCTA		

[a] For these genes, each reaction contained 10 µl of SYBR Green Master Mix (Applied Biosystems), 0.2 µM of each primer and 250 ng of cDNA in a volume of 20 µl. Reactions were run with the following conditions: 10 min at 95°C, 44 cycles of 15 s at 95°C and 1 min at 60°C, followed by a melting curve program (60-95°C) implying temperature increases of 1°C each minute.

[b] For these genes, each reaction contained 5 µl of PowerUp SYBR Green Master Mix (Applied Biosystems), 0.2 µM of each primer and 250 ng of cDNA in a volume of 10 µl. Reactions were run with the following conditions: 2 min at 50°C, 2 min at 95°C, 44 cycles of 15 s at 95°C, 15 s at 60°C and 45 s at 75°C, and a melting curve program (60-95°C) of 1°C of temperature increment for each minute.

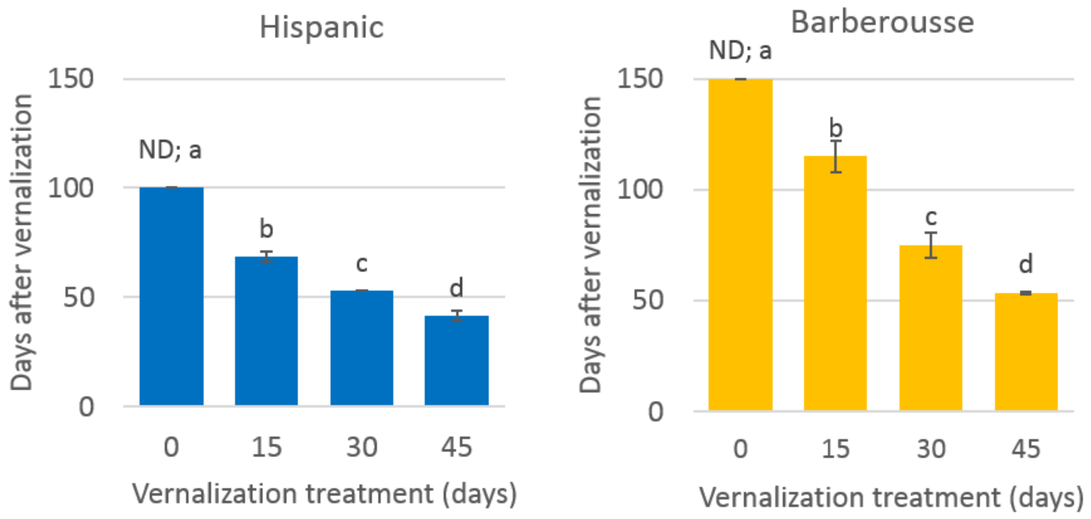

Figure S1. Flowering date under different vernalization treatments. Both varieties, ‘Hispanic’ and ‘Barberousse’, were studied independently. Hispanic’s experiment ended after 100 days, whereas Barberousse’s experiment ended 150 days after starting. ND: not determined. For each vernalization treatment, bars with different letter are significantly different at $P<0.05$ (LSD test).

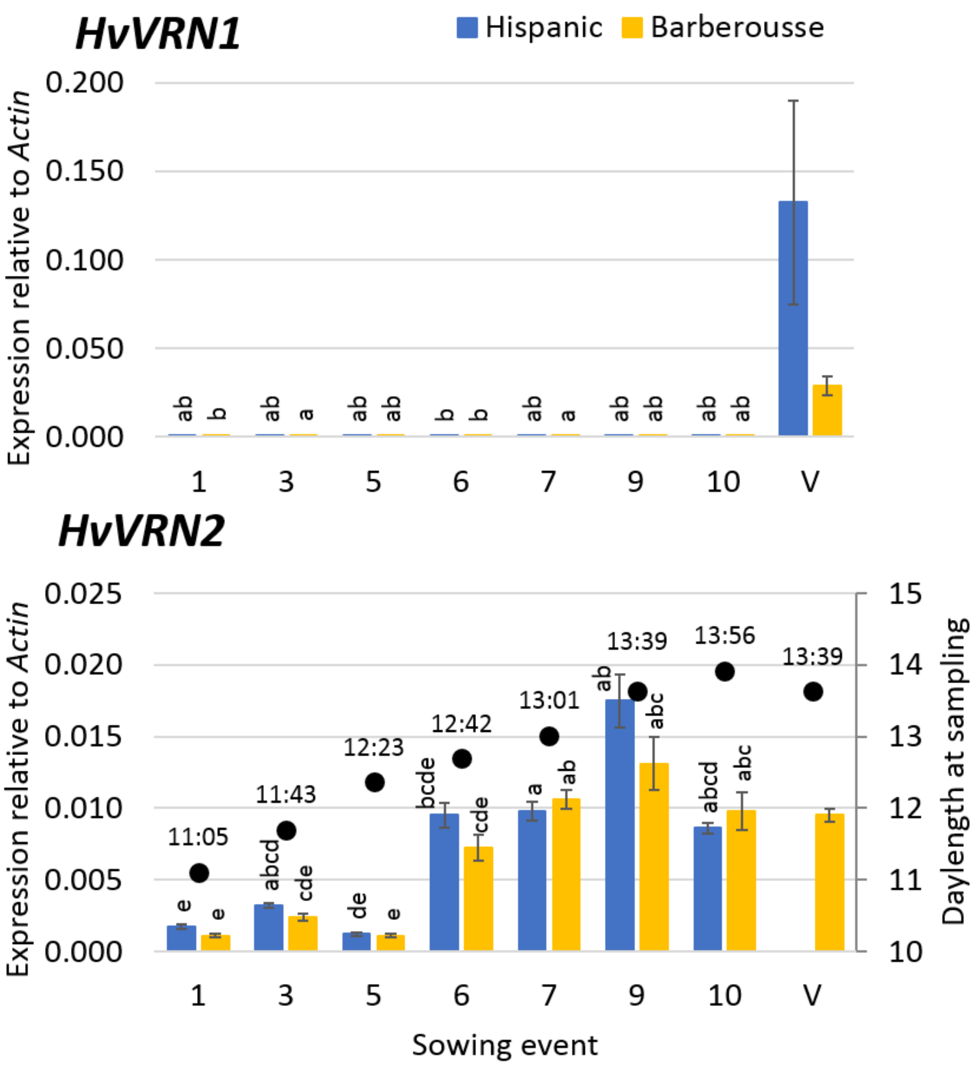

Figure S2. Gene expression in 2-week-old plants sown under natural and increasing photoperiod (without vernalization and control). X-axis represents the successive sowings, from 11th February until April 15th. Unvernalized plants (sowings 1 to 10) and vernalized control (V) of 'Hispanic' (blue) and 'Barberousse' (yellow). Black dots denote day-length at sampling date (labels in HH:MM). Mean of 3 biological replicates. Error bars are SEM. For each NV time-point, bars with the same letter are not significantly different at $P<0.05$ according to ANOVA that included genotypes and all sampling times.

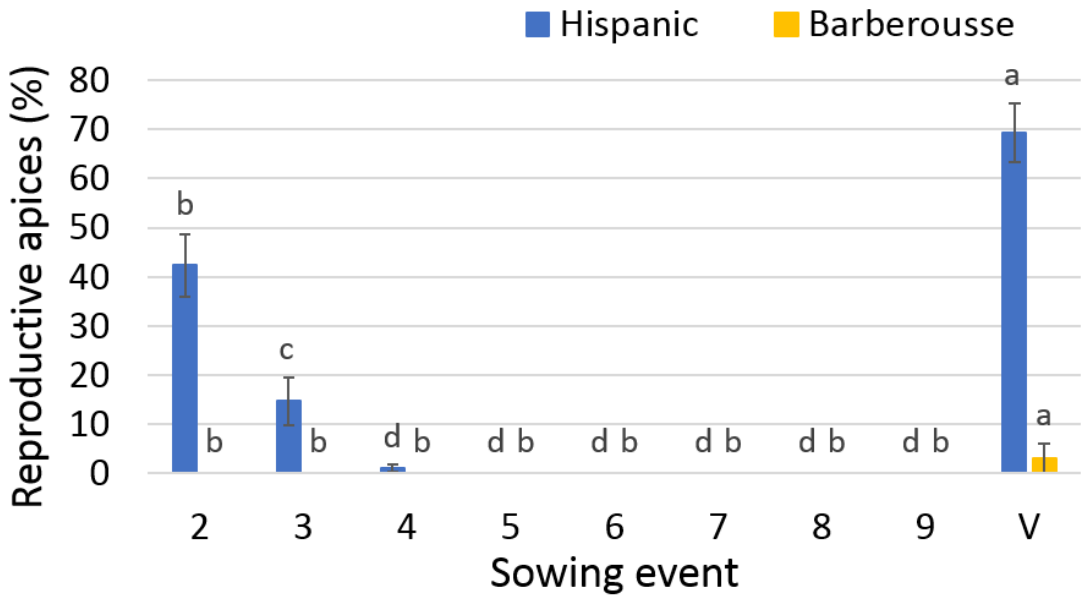

Figure S3. Percentage of reproductive apices with respect to the total (vegetative and reproductive) after 100 days of the experiment. Mean of 10-12 plants. Error bars are SD. For each variety, bars with the same letter are not significantly different at $P<0.05$ (LSD test).

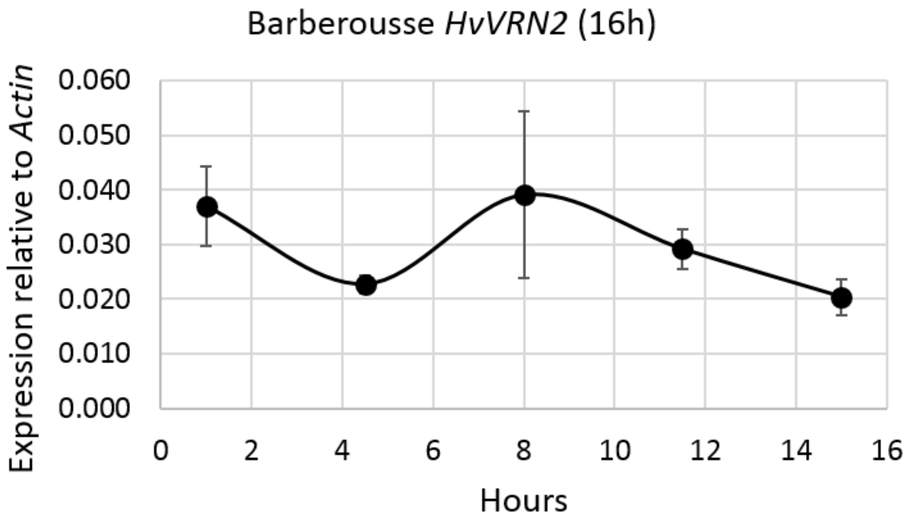

Figure S4. *HvVRN2* diurnal expression in ‘Barberousse’ unvernalized plants grown under LD (16 h light). Samples were taken at 1, 4.5, 8, 11.5 and 15 h since the start of the light period. Error bars represent standard error of mean of three biological replicates.

A) HvCO2

```
Morex_AF490470          MNCNFNSDLLEKEAGRTSFPWARPCDGCHAAPSAVYCCADAAYLCSSCDTQVHSANRVAS
Igri_AF490469           MNCNFNSDLLEKEAGRTSFPWARPCDGCHAAPSAVYCCADAAYLCSSCDTQVHSANRVAS
Barberousse_HvCO2       MNCNFNSDLLEKEAGRTSFPWARPCDGCHAAPSAVYCCADAAYLCSSCDTQVHSANRVAS
Hispanic_HvCO2          MNCNFNSDLLEKEAGRTSFPWARPCDGCHAAPSAVYCCADAAYLCSSCDTQVHSANRVAS
                        ************************************************************
                                                             B-box domain (1)

Morex_AF490470          RHERVRVCETCESTPAVLACHADAAALCTACDAQVHSANPIAQRHQRVPVLPLPAVAIPA
Igri_AF490469           RHERVRVCETCESTPAVLACHADAAALCTACDAQVHSANPIAQRHQRVPVLPLPAVAIPA
Barberousse_HvCO2       RHERVRVCETCESAPAVLACHADAAALCTACDAQVHSANPIAQRHQRVPVLPLPAVAIPA
Hispanic_HvCO2          RHERVRVCETCESAPAVLACHADAAALCTACDAQVHSANPIAQRHQRVPVLPLPAVAIPA
                        *************:**********************************************
                         (1)                    B-box domain (2)

Morex_AF490470          ASGFAEAEASVTAHGDKEGGEEVDSWLLRRNSDDNNCANKIDRYFNLVGYNMYYDNITCD
Igri_AF490469           ASGFAEAEASVTAHGDKEGGEEVDSWLLRRNSDDNNCANKIDRYFNLVGYNMYYDNITCD
Barberousse_HvCO2       ASGFAEAEASVTAHGDKEGGEEVDSWLLRRNSDDNNCANKIDRYFNLVGYNMYYDNITCD
Hispanic_HvCO2          ASGFAEAEASVTAHGDKEGGEEVDSWLLRRNSDDNNCANKIDRYFNLVGYNMYYDNITCD
                        ************************************************************

Morex_AF490470          PRPQEQYRMQEQQHVQNRYREKEGCECVVPPQVVMASEQQGSNYGTIGAGQAASVTAMAS
Igri_AF490469           PRPQEQYRMQEQQHVQNRYREKEGCECVVPPQVVMASEQQGSNYGTIGAGQAASVTAMAS
Barberousse_HvCO2       PRPQEQYRMQEQQHVQNRYREKEGCECVVPPQVVMASEQQGSNYGTIGAGQAASVTAMTS
Hispanic_HvCO2          PRPQEQYRMQEQQHVQNRYREKEGCECVVPPQVVMASEQQGSNYGTIGAGQAASVTAMTS
                        **********************************************************:*

Morex_AF490470          TYTASISNDISFSSMEVGIVPDNTRPNISNRNILTSSEAIELSGHSLQMPVHFSSMDREA
Igri_AF490469           TYTASISNDISFSSMEVGIVPDNTRPNISNRNILTSSEAIELSGHSLQMPVHFSSMDREA
Barberousse_HvCO2       TYTASISNDISFSSMEVGIVPDNTRPNISNRNILTSSEAIELSGHSLQMPVHFSSMDREA
Hispanic_HvCO2          TYTASISNDISFSSMEVGIVPDNTRPNISNRNILTSSEAIELSGHSLQMPVHFSSMDREA
                        ************************************************************

Morex_AF490470          RVLRYKEKKQARKFQKTIRYATRKAYAEARPRIKGRFAKRSDIEHEENHMLSPPALPDTS
Igri_AF490469           RVLRYKEKKQARKFQKTIRYATRKAYAEARPRIKGRFAKRSDIEHEENHMLSPPALPDTS
Barberousse_HvCO2       RVLRYKEKKQARKFQKTIRYATRKAYAEARPRIKGRFAKRSDIEHEENHMLSPPALPDTS
Hispanic_HvCO2          RVLRYKEKKQARKFQKTIRYATRKAYAEARPRIKGRFAKRSDIEHEENHMLSPPALPDTS
                        ************************************************************
                                        CCT domain

Morex_AF490470          SYNTVPWF
Igri_AF490469           SYNTVPWF
Barberousse_HvCO2       SYNTVPWF
Hispanic_HvCO2          SYNTVPWF
                        ********
```

B) HvCO9

```
Barberousse_HvCO9      MSAASGAACRVCGGGSEDCSCLLQRGRGVAAARCGVADLNRGFPGMFGQAAEEPAAVDVV
Steptoe_AB592331       MSAASGAACRVCGGGSEDCSCLLQRGRGVAAARCGVADLNRGFPGMFGQAAEEPAAVDVV
Hispanic_HvCO9         MSAASGAACRVCGGGSEDCSCLLQRGRGVAAARCGVADLNRGFPGMFGQAAEEPAAVDVV
Morex_AB592332         MSAASGAACRVCGGGSEDCSCLLQRGRGVAAARCGVADLNRGFPGMFGQAAEEPAAVDVV
                       ************************************************************

Barberousse_HvCO9      SGGGGAAAVGLQEFQFFGQEDHESVAWLFNDHAPIGGEDRLQHRSAVTEQLQRRQAFDAY
Steptoe_AB592331       SGGGGAAAVGLQEFQFFGQEDHESVAWLFNDHAPIGGEDRLQHRSAVTEQLQRRQAFDAY
Hispanic_HvCO9         SGGGGAAAVGLQEFQFFGQEDHESVAWLFNDHAPIGGEDRLQHRSAVTEQLQRRQSFDAY
Morex_AB592332         SGGGGAAAVGLQEFQFFGQEDHESVAWLFNDHAPIGGEDRLQHRSAVTEQLQRRQSFDAY
                       *******************************************************:****

Barberousse_HvCO9      AEYQPGHGLTFDVPVPVPLSRDVVDTAILGLGGGNPVTSAATIMPYCGRETLTFTEAAAS
Steptoe_AB592331       AEYQPGHGLTFDVPVPVPLSRDVVDTAILGLGGGNPVTSAATIMPYCGRETLTFTEAAAS
Hispanic_HvCO9         AEYQPGHGLTFDVPVPVPLSRDVVDTAILGLGGGNPVTSAATIMPYCGRETLTFTEAAAS
Morex_AB592332         AEYQPGHGLTFDVPVPVPLSRDVVDTAILGLGGGNPVTSAATIMPYCGRETLTFTEAAAS
                       ************************************************************

Barberousse_HvCO9      SVVDPNDDTAAGLANGGAYSAGPSGGGGVVGDVPAPTELREAKLMRYKEKRKRRRYEKQI
Steptoe_AB592331       SVVDPNDDTAAGLANGGAYSAGPSGGGGVVGDVPAPTELREAKLMRYKEKRKRRRYEKQI
Hispanic_HvCO9         SVVDPNDDTAAGLANSGAYSAGPSGGGGVVGDVPAPTELREAKLMRYKEKRKRRRYEKQI
Morex_AB592332         SVVDPNDDTAAGLANSGAYSAGPSGGGGVVGDVPAPTELREAKLMRYKEKRKRRRYEKQI
                       ***************.********************************************
                                                                  CCT domain (continued)

Barberousse_HvCO9      RYASRKAYAEMRPRVKGRFAKVPDGGEGAAPSPPQQPTQAAGYEPSRLDLGWFRS
Steptoe_AB592331       RYASRKAYAEMRPRVKGRFAKVPDGGEGAAPSPPQQPTQAAGYEPSRLDLGWFRS
Hispanic_HvCO9         RYASRKAYAEMRPRVKGRFAKVPDGGEGAAPSPPQQPTQAAGYEPSRLDLGWFRS
Morex_AB592332         RYASRKAYAEMRPRVKGRFAKVPDGGEGAAPSPPQQPTQAAGYEPSRLDLGWFRS
                       *******************************************************
                           CCT domain
```

C) HvOS2

```
Morex_HORVU3Hr1G095240.6    MARRGRVELRRIEDRTSRQVRFSKRRSGLFKKAFELSVLCDAEVALLVFSPAGRLYEYAS
Hispanic_HvOS2              MARRGRVELRRIEDRTSRQVRFSKRRSGLFKKAFELSVLCDAEVALLVFSPAGRLYEYAS
Barberousse_HvOS2           MARRGRVELRRIEDRTSRQVRFSKRRSGLFKKAFELSVLCDAEVALLVFSPAGRLYEYAS
Zangqing320                 MARRGRVELRRIEDRTSRQVRFSKRRSGLFKKAFELSVLCDAEVALLVFSPAGRLYEYAS
Igri_HM130526               MARRGRVELRRIEDRTSRQVRFSKRRSGLFKKAFELSVLCDAEVALLVFSPAGRLYEYAS
                            ************************************************************
                                                MADS-box domain (continued)

Morex_HORVU3Hr1G095240.6    SSIEGTYDRYQAFAGAGKDVSEGGASNNNVNDPSNIQSRLKDITSWSLQNNADDSDANEL
Hispanic_HvOS2              SSIEGIYDRYQAFAGAGKDVSEGGASNNNDGDPSNIQSRLKDITSWSLQNNADDSDANEL
Barberousse_HvOS2           SSIEGTYDRYQAFAGAGKDVSEGGASNNNDGDPSNIQSRLKDITSWSLQNNADDSDANEL
Zangqing320                 SSIEGTYDRYQAFAGAGKDVSEGGASNNNDGDPSNIQSRLKDITSWSLQNNADDSDANEL
Igri_HM130526               SSIEGTYDRYQAFAGAGKDVSEGGASNNNDGDPSNIQSRLKDITSWSLQNNADDSDANEL
                            ***** *********************** .*****************************
                              MADS-box domain

Morex_HORVU3Hr1G095240.6    VKLEKLLTDALKKTKSKKILAQRNSGAGTIASGENSRRF
Hispanic_HvOS2              VKLEKLLTDALKKTKSKKILAQRNSGAGTSASGENSRRF
Barberousse_HvOS2           VKLEKLLTDALKKTKSKKILAQRNSGAGTIASGENSRRF
Zangqing320                 VKLEKLLTDALKKTKSKKILAQRNSGAGTIASGENSRRF
Igri_HM130526               VKLEKLLTDALKKTKSKKILAQRNSGAGTSASGENSRRF
                            ***************************** *********
```

Figure S5. Alignments of (A) HvCO2, (B) HvCO9, and (C) HvOS2 predicted proteins. Protein domains for HvCO2 [10]; HvCO9 [Prosite v20.79 (http://prosite.expasy.org/)], and HvOS2 [6] are indicated.

References

1. Griffiths S, Dunford RP, Coupland G, Laurie DA. The Evolution of *CONSTANS*-Like Gene Families in Barley, Rice, and Arabidopsis. Plant Physiol. 2003;131 April:1855–67.
2. Kikuchi R, Kawahigashi H, Oshima M, Ando T, Handa H. The differential expression of *HvCO9*, a member of the *CONSTANS*-like gene family, contributes to the control of flowering under short-day conditions in barley. J Exp Bot. 2012;63:773–84.
3. Yan L, Fu D, Li C, Blechl a, Tranquilli G, Bonafede M, et al. The wheat and barley vernalization gene VRN3 is an orthologue of FT. Proc Natl Acad Sci. 2006;103:19581–6.
4. Kikuchi R, Kawahigashi H, Ando T, Tonooka T, Handa H. Molecular and functional characterization of PEBP genes in barley reveal the diversification of their roles in flowering. Plant Physiol. 2009;149:1341–53.
5. Faure S, Higgins J, Turner A, Laurie DA. The *FLOWERING LOCUS T*-like gene family in barley (*Hordeum vulgare*). Genetics. 2007;176:599–609.
6. Greenup AG, Sasani S, Oliver SN, Talbot MJ, Dennis ES, Hemming MN, et al. *ODDSOC2* is a MADS box floral repressor that is down-regulated by vernalization in temperate cereals. Plant Physiol. 2010;153:1062–73.
7. Hemming MN, Peacock WJ, Dennis ES, Trevaskis B. Low-temperature and daylength cues are integrated to regulate FLOWERING LOCUS T in barley. Plant Physiol. 2008;147:355–66.
8. Trevaskis B, Hemming MN, Peacock WJ, Dennis ES. *HvVRN2* responds to daylength, whereas *HvVRN1* is regulated by vernalization and developmental status. Plant Physiol. 2006;140:1397–405.
9. Waddington SR, Cartwright PM. A quantitative Scale of Spike Initial and Pistil Development in Barley and Wheat. Ann Bot. 1983;51:119–30.
10. Cockram J, Thiel T, Steuernagel B, Stein N, Taudien S, Bailey PC, et al. Genome Dynamics Explain the Evolution of Flowering Time CCT Domain Gene Families in the Poaceae. PLoS One. 2012;7:e45307.
